# UShER-TB: scalable, comprehensive, accessible phylogenomic analysis of Mycobacterium tuberculosis

**DOI:** 10.1099/mgen.0.001859

**Published:** 2026-10-22

**Authors:** Lily M. Karim, Francisco José Martínez-Martínez, Ash O’Farrell, Angie S. Hinrichs, Theo Sanderson, Zamin Iqbal, Varvara K. Kozyreva, John M. Bell, Mariana G. López, Iñaki Comas, Russell Corbett-Detig

**Affiliations:** 1 Department of Biomolecular Engineering, University of California, Santa Cruz, USA; 2 Genomics Institute, University of California, Santa Cruz, USA; 3 Tuberculosis Genomics Unit, Instituto de Biomedicina de Valencia (IBV), CSIC, Valencia, Spain; 4 Department of Infection Biology, London School of Hygiene and Tropical Medicine, London, UK; 5 Milner Centre for Evolution, University of Bath, Bath, UK; 6 Microbial Diseases Laboratory, Infectious Diseases Laboratories Division, Center for Laboratory Sciences, California Department of Public Health, Richmond, CA, USA; 7 CIBER in Epidemiology and Public Health, Madrid, Spain

**Keywords:** bioinformatics, computational biology, infectious disease, phylogenetics, tuberculosis, workflow

## Abstract

**Background.** *Mycobacterium tuberculosis*, the bacterium responsible for tuberculosis, remains a leading cause of global infectious disease deaths. While genomic epidemiology is essential for understanding transmission dynamics and the rise of antibiotic resistance, significant computational limitations currently prevent comprehensive phylogenetic analysis of the vast number of publicly available *M. tuberculosis* genomes.

**Methods.** We developed UShER-TB, a comprehensive collection of community resources, including a computational analysis pipeline designed for scalable phylogenomic analysis of *M. tuberculosis*. To demonstrate this system’s utility, we processed 129,312 genomes to construct a global phylogeny. We evaluated the system’s accuracy in transmission cluster reconstruction, its ability to identify novel genetic lineages and its performance in placing ancient DNA samples within the global tree.

**Results.** The pipeline successfully constructed a global phylogeny capturing genomic diversity from over 129,000 samples. UShER-TB achieved high accuracy in reconstructing transmission clusters, reaching an adjusted Rand index of 0.95 when compared to conventional phylogenomic methods. This tool also facilitated the identification of putative novel lineages and sublineages. Furthermore, the platform enabled the successful phylogenetic placement of ancient samples. The system allows for real-time analysis of new genomes to reveal transmission dynamics and introduction patterns on a global scale.

**Conclusions.** This approach overcomes longstanding computational barriers in genomic surveillance. UShER-TB provides researchers with efficient tools for tuberculosis monitoring, particularly for resource-limited settings where the disease burden is highest.

**Trial registration.** Not applicable.

**Impact Statement**

The development of UShER-TB addresses the critical computational bottleneck in *M. tuberculosis* genomic epidemiology, successfully enabling rapid phylogenetic placement of over 129,000 genomes. By providing a scalable platform that maintains high topological accuracy while identifying novel lineages and transmission dynamics, this study demonstrates a shift from retrospective data collection to real-time genomic surveillance. This infrastructure is essential for global health efforts, offering a practical and efficient tool for monitoring tuberculosis transmission in resource-limited settings where the disease burden is most severe.

## Availability of Data and Materials

### Code availability

We provide major workflow components to support our conclusions and enable future applications here. The sample processing pipeline, myco, has components that are designed to be modular (such as SRANWRP, which pulls FASTQs from SRA) and may be useful for analysis of other organisms. Downsampling, masked-to-reference regions, decontamination, QC cutoffs and more can be adjusted by myco users who may wish to use the overall pipeline but with different metrics than what we used for this analysis; see myco’s documentation (https://github.com/aofarrel/myco) for more information.

Two versions of the myco pipeline are available – myco_raw, which takes in FASTQ as direct file inputs, and myco_sra, which takes in a text file of BioSample accessions from which FASTQ are downloaded. myco_sra and myco_raw share a GitHub repository at https://github.com/aofarrel/myco and have Dockstore entries at https://dockstore.org/workflows/github.com/aofarrel/myco/myco_sra and https://dockstore.org/workflows/github.com/aofarrel/myco/myco_raw, respectively.

usher-sampled and matUtils [including the local masking function (see Methods)] are publicly available at https://github.com/yatisht/usher.

Ranchero is available on PyPI (https://pypi.org/project/ranchero/). Ranchero’s source code and extensive documentation are available at https://github.com/aofarrel/ranchero.

DOI versions of these WDL workflows are also available:

- myco_sra: https://doi.org/10.5281/zenodo.15080116
- myco_raw: https://doi.org/10.5281/zenodo.15061623

Custom scripts for analysis and visualizations presented in this work are available via GitHub at https://github.com/lilymaryam/Mtb_global_phylogeny.

### Data Availability

All genotypic data used in this work are available from the Sequence Read Archive. See Table S24, available in the online Supplementary Material, for detailed information about specific BioSamples.

As described in our Methods, metadata – including phenotypic drug susceptibility testing (pDST), pipeline outputs and sample collection information – was sourced from a variety of open sources. We used Ranchero to standardize and compile all gathered metadata in TSV format, available in Table S3.xlsx.tsv. Note that this file is derived from a very wide BigQuery search of SRA, as described in Text S1, and includes many samples not present on the tree. A version of our metadata subset only to samples on the tree can be found in Table S24.xlsx.

The tree, annotated with a subset of our metadata, may be viewed using Taxonium: https://taxonium.org/tuberculosis/SRA. The tree may be downloaded from our supplemental drive at https://drive.google.com/drive/folders/1Tzzmh3Y1WXe_cLmdHZIToppyGvfvJqNL or from https://hgdownload.gi.ucsc.edu/hubs/GCF/000/195/955/GCF_000195955.2/UShER_Mtb_SRA/.

All supplemental files are available on FigShare https://doi.org/10.6084/m9.figshare.32896928 [1].

### Reproducibility

We have provided Table S22 which contains the versions of all the tools we used for our analyses. We have provided Table S23 and Text S9 which explain the computational resources used, as well as additional details for running myco and usher-sampled, the most computationally intensive tools of this analysis. We have provided commands and instructions in the README of each directory in the GitHub (https://github.com/lilymaryam/Mtb_global_phylogeny) which corresponds to the different analyses in the manuscript.

## Background

Tuberculosis (TB) has been present in human and animal populations for thousands of years. The first solid evidence of the *Mycobacterium tuberculosis* (MTB) pathogen infecting humans dates back at least 8,000 years [2]. Since then, TB has probably killed more people than any other single pathogen [3]. It has been the leading cause of death from a single infectious agent worldwide for decades, only briefly surpassed by COVID-19 [4], and is one of the top ten leading causes of mortality [4, 5]. It is estimated that there are 10 million new TB cases and more than 1.2 million deaths annually [4, 5], disproportionately affecting disadvantaged populations. Its burden varies with factors such as overcrowding, HIV status, malnutrition, diabetes and access to healthcare [4].

Novel genomic tools improve strategies to halt transmission locally and globally. Global strategies implemented in the 1990s such as directly observed therapy [6, 7] have been successful in reducing deaths from a peak of over 2 million deaths per year. However, the decline in the number of cases has been slow (at a rate of 2% per capita), and the world population growth counteracts this decrease such that overall case numbers remain relatively steady [8]. Current progress is therefore far from the World Health Organization Sustainable Development Goals’ target of ending the TB epidemic by 2030. Transmission remains common even in low-burden countries. An additional issue is the emergence of drug-resistant TB. While global rates are low (~3%), some regions report that more than 20% of new cases are multidrug-resistant, implying the transmission of already drug-resistant strains [9]. Recent reports also show substantial transmission of strains resistant to new drugs recently introduced in treatment programmes [10]. Genomic techniques developed in recent years have proven essential to understanding the nature of TB transmission, quantifying TB transmission in different settings, identifying transmission dynamics, enabling outbreak investigations, differentiating between relapse and reinfection and distinguishing imported versus local cases [11]. In addition, genomic data fosters biological research by allowing us to understand the evolution of the pathogen, its phylogeographic spread and its co-evolution with human populations [12].

The large MTB genome size, with 4.4 million base pairs, makes these analyses very time-consuming as the number of samples increases. With current tools, phylogenetic inference can take several days to finish with just a few thousand samples, but more than 250,000 short-read MTB datasets are publicly available on the Sequence Read Archive (SRA). This means that most available data must be omitted from analyses and chosen for inclusion *a priori*, even though this may accidentally exclude relevant data. Although sample selection is performed based on scientific evidence (e.g. previous epidemiological studies), this potential bias may obscure new insights into transmission.

During the COVID-19 pandemic, the extraordinary pace and scale of generation of SARS-CoV-2 genome sequences inspired the creation of phylogenetic tools like MAPLE (MAximum Parsimonious Likelihood Estimation [13]) and UShER (Ultrafast Sample placement on Existing tRees [14, 15]). The UShER tool accepts a pre-existing phylogeny and places new sequences on it, avoiding the need to reconstruct the whole tree from scratch when new samples are analysed. UShER was successfully used in regional and local genomic epidemiology studies [16, 17] and is currently the tool used to identify SARS-CoV-2 lineages globally as a part of the Pangolin network [15]. Open-source tools like UShER, which can be used universally and are accessible, are relevant for closing the gaps in analytical capacities between the Global South and the Global North [18]. This is particularly true for TB, where most of the burden is concentrated in the poorest socioeconomic regions of the world [19].

UShER makes it possible to reconstruct massive phylogenies for SARS-CoV-2 with potential applications to diverse other pathogens. Here, we expand this platform to MTB, whose genome is ~150× the size of SARS-CoV-2. Using UShER to study MTB shows that it is a valuable tool for genomic epidemiology studies in bacteria: (1) it has the potential to use most publicly available data, (2) complex computational analysis is handled in a user-friendly way, and (3) it is fast and efficient, thereby enabling application in resource-limited settings and offering rapid results. In this study, we detail (1) the new workflows developed to adapt UShER for MTB genomic epidemiology, (2) the creation of a comprehensive UShER online phylogenomics platform, including 129,312 available MTB samples, and (3) results obtained from UShER for transmission reconstruction, surveillance, analysis of ancient DNA samples and potential for public health applications. Hereafter, we refer to the complete product, workflows, new methods and resulting datasets as ‘UShER-TB’ (Table S1.xlsx). Collectively, this work develops tools and resources to build trees with as many MTB samples as needed in an efficient way and to place new samples in those trees, enabling efficient phylogenetic and transmission studies at scale.

## Methods

### Data acquisition

We considered almost all publicly available Illumina-processed MTBC (*Mycobacterium tuberculosis* complex) sequences on the SRA [20] as of June 2024, which we queried using the SRA Cloud-based Metadata Table (https://www.ncbi.nlm.nih.gov/sra/docs/sra-cloud-based-metadata-table/); see Text S1for the exact query.

### Metadata processing

We curated metadata from fields associated with each sample’s SRA entry, or in some cases, by parsing supplemental material of publications. In particular, we obtained sample metadata through direct parsing of NCBI fields, pulled using BigQuery (https://www.ncbi.nlm.nih.gov/sra/docs/sra-bigquery-examples/), existing publications (Table S2.xlsx) and expert consultation (E. Roycroft and J. Andrews pers comm.). We note that there is a wide variety of metadata fields on SRA, which are not always consistent. Other issues can arise when comparing across samples that use different formats for date of isolation or older taxonomic/lineage systems. To aid in this, as well as to harmonize metadata parsed from individual publications, we developed an auxiliary tool called Ranchero (https://github.com/aofarrel/ranchero). Although designed for wrangling mycobacterial data, most of Ranchero’s functions are compatible with other organisms. Details about how Ranchero adjudicates conflicting metadata fields can be found in Ranchero’s documentation, available on its GitHub page.

### Sample processing

We passed sample accessions to our MTBC processing pipeline, myco (https://dockstore.org/workflows/github.com/aofarrel/myco/myco_sra), which extracts sequence data from the SRA as paired-end FASTQ files and generates variant information. myco is written as a Workflow Description Language (WDL) [21] pipeline for machines capable of running Docker, as well as the cloud compute platform Terra [22], the latter of which we used for this part of the project. The myco pipeline uses SRANWRP (https://github.com/aofarrel/sranwrp), a wrapper for sra-tools [23] that we developed to streamline the mass download of BioSamples that include at least one valid Illumina paired-end sample, while also automating the exclusion of entries with incompatible data formats or corrupted FASTQ files. The myco pipeline also uses clockwork [24, 25], a CRyPTIC consortium [26] data processing pipeline, for accurate genotyping of short-read sequencing of TB samples [25, 27–30].

We downsampled FASTQ files above 450 megabytes to 1,000,000 reads using seqtk sample [31] and discarded samples with fewer than 1,000 reads. We masked to reference regions of the genome that are known to be difficult to genotype [32] using the CRyPTIC mask file [26, 33]. Our pipeline converts low-depth regions and indel/structural variants to ambiguous nucleotides, so that neither is used for phylogenetic placement, and performs several quality control (QC) checks (see details in https://github.com/aofarrel/myco/tree/main/doc).

For each QC-passing SRA BioSample, version 6.2.4 of myco generated a clockwork [25]-processed bedGraph [34] coverage file and VCF [35] (Variant Call Format), the latter of which is converted into MAPLE file format [13] using the genome H37Rv (NC_000962.3) as the reference. The MAPLE file format [13] is used for downstream analyses because VCF is less efficient for genomic epidemiological applications at this scale [13]. Our pipeline produces TBProfiler [36, 37] (version 4.4.2 for this analysis; updated versions have since been integrated into myco) reports, which provide lineage designations and predicted drug resistance for each sample. These reports are parsed and used as metadata for the final tree, along with the other metadata standardized by Ranchero (Table S3.xlsx; see Results).

### Phylogenetic inference

A total of 135,180 MTB samples were processed with the myco pipeline and output in MAPLE file format [13]. These samples were concatenated into a single MAPLE file which was then input along with the H37Rv *M. tuberculosis* reference genome (NC_000962.3) into the usher-sampled subtool [15] of UShER (version 0.6.4) to infer and optimize a global MTB phylogenetic tree with default parameters on a 64-thread, 1 TB RAM Linux server (see usher-sampled commands in GitHub README https://github.com/lilymaryam/Mtb_global_phylogeny/tree/main and source code https://github.com/yatisht/usher). The inferred phylogenetic tree is output in a Mutation Annotated Tree (MAT) protocol buffer (.pb) file format [14]. We used TaxoniumTools [38] (PyPi version 2.0.86) subtool usher_to_taxonium to convert the MAT file format to a json format compatible with Taxonium.org [38] (jsonl.gz) for visualizing the tree.

### Rerooting and pruning *Mycobacterium canettii*

To reroot our global phylogeny, we first identified the *Mycobacterium canettii* clade [39]. Though *M. canettii* is a member of the MTBC, it has significantly different biological properties and is often used as an outgroup [40–44]. We rooted the tree at the parent node of lineage 8 and *M. canettii*, which represented a common ancestor between *M. canettii* and the rest of the bacterial complex, and placed a leaf node with a branch length of 0 as the child of this identified root to track this ancestral node in the final tree. We then pruned the 137 *M*. *canettii* samples (Table S4.xlsx) leaving a phylogeny with 135,043 samples.

### Identification of potential coinfections

After inferring the initial global phylogeny and pruning *M. canettii* to reach a 135,043-sample tree, we identified and removed samples putatively coinfected with two or more genetically distinct strains. We used the VCFs generated from myco to obtain adjudicated variant calls and fraction of read support (FRS) information [25]. FRS indicates the percentage of reads that support a given variant call. Because ambiguous nucleotides and missing data are preferentially resolved to reference by usher-sampled when more than one equally parsimonious assignment is possible [14], coinfected samples are expected to be placed near to the common ancestor of coinfecting lineages. For each VCF, we calculated the percentage of variants that were classified as having a low FRS value (<0.85) (Table S5.xlsx). We identified all samples where greater than 19% (>0.19) of their variants were classified as having a low FRS (<0.85) value and labelled these samples as ‘candidate coinfections’ (Table S6.xlsx). Our approach for identifying 19% as the reasonable appropriate threshold is described in (Text S2). Our analysis of dataset size and cluster recovery across alternate thresholds is available in Table S7 and Fig. S1. With this threshold, we identified and removed 5,631 samples (Table S6.xlsx). We then visually inspected the global phylogeny and found that many but not all of the ancestrally placed samples were removed by this screening.

We performed a final manual pruning step to identify ancestrally placed samples that were not removed by automated QC. Candidates for pruning were first identified by visual inspection of the phylogeny. We flagged samples that were placed basally relative to their broader clade, as these sample placements suggest artifactual or ancestral placement inconsistent with their metadata. Flagged samples were then assessed against two confirming criteria: whether the sample was isolated with no neighbouring sequences in close proximity and whether it exhibited a terminal branch length of zero, indicating placement at an internal node rather than a distinct tip. One hundred samples met these criteria and were pruned prior to downstream analysis (Table S8.xlsx, Fig. S2).

### Post-inference local masking

Missing sequence information in the genomic dataset can cause issues during phylogenetic reconstruction. Missing data can obscure true genetic similarity between samples, especially when neighbouring samples have mutations at those sites. This can lead placement algorithms to overestimate their differences. To mitigate this, we mask such positions to reduce the impact of misleading signals. We describe these issues in Fig. 3(a), with an example phylogeny. We developed an approach that reduces the impact of missing data on phylogenetic reconstruction (see Fig. 3a) that we call post-placement local masking. Our method searches the tree for sample pairs within a nominal ‘masking distance’, which is their phylogenetic distance in SNPs. It then identifies all missing positions in a given sample and masks those sites in all other samples within the masking distance. By looking only within other closely related samples, we are able to mask locally. We then use matOptimize [45] to rearrange the tree to optimal parsimony after masking. Our masking tool is available in the matUtils library [46]. This method may improve the analysis of transmission between closely related samples because it does not arbitrarily resolve genotypes at missing sites.

### Evaluation of the accuracy of UShER-TB for public health inquiry

We simulated a 30,000-sample MTB genomic dataset, constructed a phylogeny and evaluated UShER-TB’s accuracy. To do this, we used Alisim [47] to simulate a large genomic dataset of MTB genomic samples. We extracted a subtree of 30,000 neighbouring samples from the inferred global phylogeny (see above) and input this subtree as the guide tree in Alisim (IQ-TREE 2 v2.2.0) (hereafter the ‘truth tree’), the length of the H37Rv (NC_000962.3) genome as alignment length and the General Time Reversible [48] model with MTB base frequencies (A:0.18, C:0.32, G:0.32, T:0.18) as our evolutionary model. We emphasize that although the tree based on real data must contain inference errors, once we fix a topology as truth for simulation of genomic data, those errors are no longer relevant. After we reconstructed the phylogeny using usher-sampled [15], we calculated the normalized Robinson–Foulds distance [49] between the truth tree and the inferred tree after collapsing internal nodes that do not contain mutations (as in Kramer *et al*. [50]). This approach is appropriate because some nodes are multifurcating and would be arbitrarily resolved in binary trees, which is typical in densely sampled genomic epidemiology applications [50–52].

We also evaluated the accuracy of sample placements in relation to their closest neighbours, as a typical public health analysis goal is to find all known samples with a recent shared common ancestor. We devised a test that computes the percentage of the truth neighbours that can be recovered within a specified distance of the target sample in the inferred tree. We extracted neighbouring samples from each tested sample in the truth tree based on the expected maximum age of divergence of neighbouring samples (i.e. a 2-year divergence neighbourhood is expected to contain samples within 2 SNPs of our target [53–57]). For the analysis, we extracted truth neighbourhoods with divergence ages of 1 year, 3 years, 5 years, 8 years and 10 years. In the inferred tree, we extracted SNP neighbourhoods of distances 1, 5 and 10 from around the target sample because they are standard transmission cluster sizes that epidemiologists use for analysis [11, 54–56, 58]. For each divergent neighbourhood, we calculated the proportion of true nearest neighbours in the inferred tree, which we call the Neighbourhood Recovery Rate or NRR (Fig. S3).

### Sensitivity of cluster assignment based on local masking and FRS threshold

To test the sensitivity of final placements and cluster assignments to bioinformatic thresholds, we randomly selected 500 samples from the 129,312-sample final dataset and evaluated neighbourhood recovery across a range of FRS thresholds and local masking distances. The baseline tree for this analysis is the 135,043-sample tree composed of samples that passed through myco QC and *M. canettii* filtering. For each selected sample, we identified all neighbours within ten SNPs in the baseline tree to define a ten-SNP transmission cluster, a threshold commonly used in epidemiological research. We tested masking distances of 0, 2, 4, 6, 8 and 10 SNPs and FRS thresholds of 0.05, 0.1, 0.12–0.20 (in steps of 0.01), 0.25, 0.3, 0.5 and 0.9, spanning a wide range with finer resolution in the 0.1–0.2 region where the proportion of samples pruned changes rapidly, and therefore, finer-grained resolution is informative. At FRS 0.05, 113,013 samples are removed, leaving 22,032; at FRS 0.9, no samples are removed. For each FRS threshold, samples exceeding that value were pruned, the remaining tree was locally masked at each distance, and 10-SNP neighbourhoods were re-extracted for the 500 samples. Neighbourhood recovery was calculated as described in Methods.

### Estimating the number of introductions

To find novel introductions into specific geographic regions, we used matUtils [59]. We used matUtils’ annotate function to add lineage metadata to the global phylogeny (from Table S9.xlsx). We then ran matUtils introduce on the annotated phylogeny, which identifies all putative geographical introductions, with default settings and a metadata file associating each sample with its associated three-letter ISO code (International Organization for Standardization) (Table S10.xlsx). We separated the introduction count per country into multiple transmissions and single introductions and stratified these categories by lineage (Fig. S4).

### Sensitivity analysis for inference of introductions

We developed a simple sensitivity analysis of introduction inferences to assess the robustness of phylogeographic origin inferences to sampling variation among regions. We first identified the seven countries with the highest sample counts in the dataset: UK (GBR, *n*=14,724), China (CHN, *n*=9,780), India (IND, *n*=7,083), Peru (PER, *n*=6,795), South Africa (ZAF, *n*=6,488), Australia (AUS, *n*=4,940) and the USA (*n*=4,635) [Table S24.xlsx (metadata for the 129,312-sample final dataset)]. We conducted downsampling using 2 approaches across a range of 10–60%: (1) each country independently and (2) all countries combined, resulting in 48 phylogenetic trees total (7 countries×6 downsample levels+6 combined scenarios).

For each downsampled tree, we used the same introduction estimation method used in the Methods section, ‘Estimating the number of introductions’, and compared the results to the introduction inference performed on the full global tree. To determine how introduction counts change, we identified how the number of counts changes for each of the seven countries across all downsamplings (Fig. S5).

For each region, we identified the most common geographic origin for a new introduction in the full baseline tree. We then compared this to the most common origin for each region in each of the 48 downsampled trees. Origin stability was quantified as the percentage of downsampling scenarios in which a region’s predominant source location shifted relative to the baseline. For example, if Australia’s most common origin changed in 10 of the 48 scenarios, it would have an origin instability of 21%. We examined the relationship between sampling density (percentage of total dataset per country) and origin stability using scatter plot visualization. The five countries with the highest origin instability were also identified and annotated (Fig. S6).

### Identification of potential phylogenetically distinct clades

We first identified candidate phylogenetically distinct clades through visual inspection of the 129,312-sample tree. For each lineage, we searched for unannotated samples that were placed basally. To rule out the possibility that these samples’ unusual phylogenetic placements are due to a mixed infection, we calculated the path lengths from internal node to tip for every sample descending from the most recent common ancestor (MRCA) of these samples and samples in the sister clade (in both cases, the parent of the sample was the MRCA). We compared these distributions with a two-sided Mann–Whitney U test to determine if the branch lengths are significantly different between all descendant samples. While the mixed-infection screen does not itself prove that these samples are phylogenetically distinct, it does reveal that they were not excluded based on mixed-base criteria, making coinfection or contamination an unlikely explanation for their unusual phylogenetic positions.

### Transmission cluster assignment in local and global datasets

TB transmission surveillance is key for public health systems to evaluate transmission burden and to investigate outbreaks. In this context, we assessed the capacity of usher-sampled to reconstruct known transmission clusters, defined as groups of MTB samples with a pairwise genetic distance of a maximum of ten SNPs. We chose this because it is a common SNP threshold in MTBC cluster analysis [60].

To benchmark the robustness of a standard, widely used pipeline for cluster identification (available at https://gitlab.com/tbgenomicsunit/ThePipeline) based on pairwise distances and assignment using usher-sampled, we used a dataset from the Valencian Region in Spain (*n*=1540). We used the standard approach for cluster identification using a ten-SNP threshold and compared them to the clusters obtained from the usher-sampled phylogeny. The non-matching clusters were visually inspected to identify potential sources of discrepancy between both approaches. A matching cluster is defined as a transmission group inferred by both clustering methods with exactly the same sequences, whereas a non-matching cluster is a transmission group inferred by one method that is not found using the other since the samples are slightly different, distributed in different clusters or not in transmission. Additionally, we calculated the precision, recall and Rand index for cluster assignment to evaluate the robustness of our method against the gold standard. The R scripts used for these analyses are available at https://github.com/lilymaryam/Mtb_global_phylogeny.

After validating the use of usher-sampled to identify transmission groups, we evaluated the approach in two different scenarios. The first scenario is whether sequences from a local setting will be analysed alone as part of ongoing surveillance programmes in a country. The second scenario is when those sequences are incorporated into a large global reference phylogeny. In both cases, we evaluated the accuracy of phylogenetic placement using usher-sampled and an existing input phylogeny. Three settings with different sampling schemes, burden and transmission patterns were chosen: the aforementioned Valencian Region in Spain, a low-burden setting (<10 cases per 100,000 population, collected in the period 2014–2019, *n*=1540), Chiang Rai province in Thailand (157 cases per 100,000 population in 2023, collected in the period 1999–2011, *n*=1167) and Moldova (74 cases per 100,000 population, collected between January 2018 and December 2019, *n*=1925).

To generate a ground truth for each of the settings, we built a local and a global phylogeny (Table S11.xlsx) using usher-sampled for *de novo* phylogenies. Then, using a custom script in R (https://github.com/lilymaryam/Mtb_global_phylogeny), we obtained the pairwise genetic distance between all samples and used this matrix as input for another custom Python script (https://github.com/lilymaryam/Mtb_global_phylogeny) that obtains the clusters for a given SNP threshold, ten in our case, which was the basis for our comparison. To test whether sample placement can assign samples consistent with known transmission clusters, we removed some of the samples from each of the datasets. In the Valencian Region (*n*=1540), collected between 2014 and 2019, we removed the samples collected after 2016 (*n*=743). In Thailand (*n*=1167), collected between 1999 and 2011, and Moldova (*n*=1925), collected between 2008 and 2011, we removed 40% of the samples (*n*=467 and *n*=770, respectively) by sorting all samples by collection date and excluding the most recently collected ones. A scheme on how each dataset is compared to the ground truth can be found in Fig. 1.

**Fig. 1.**
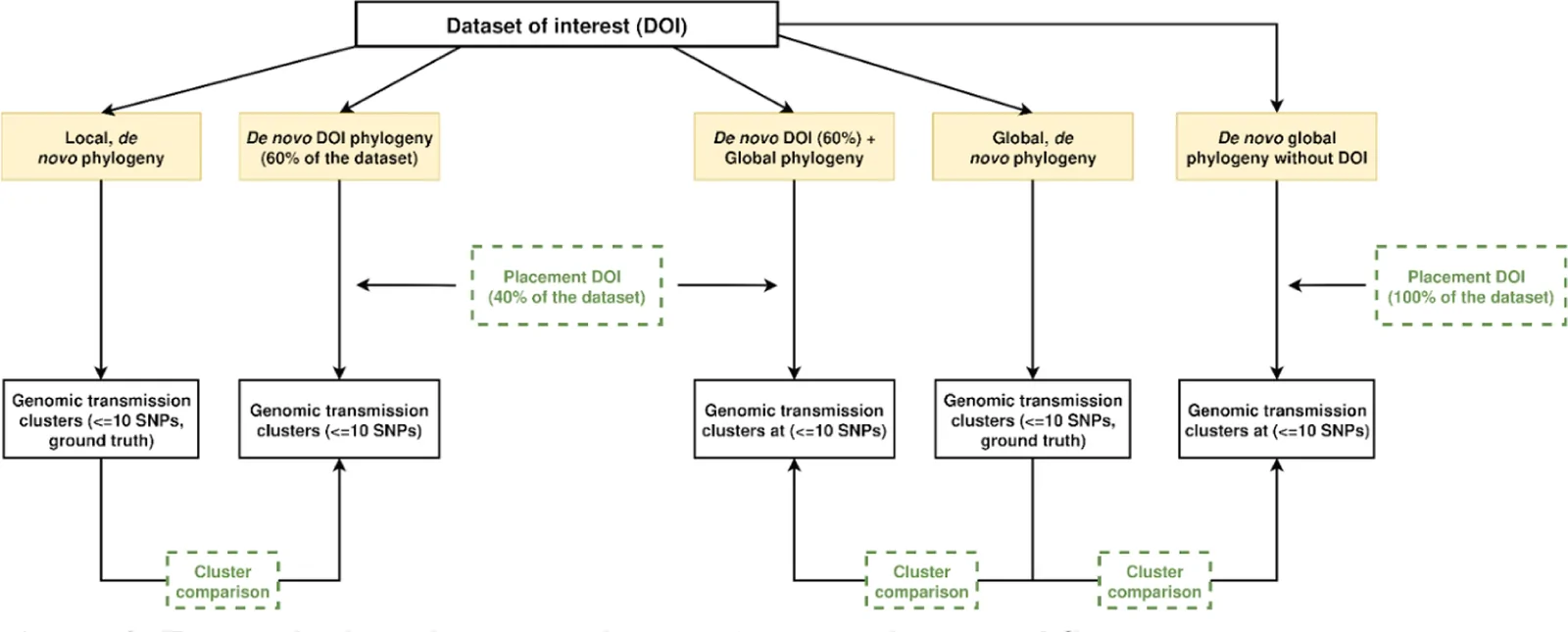
Transmission cluster assignment comparison workflow.

### Ancient MTBC genome placement with usher-sampled

We used usher-sampled to place six ancient DNA genomes from three different studies and timeframes. Among 14 genome sequences from Hungarian mummies dating back to the eighteenth century, we chose those 2 with the highest coverage and no signature of mixed infection [61]. We also included a recently sequenced MTB genome obtained from skeletal remains of a Swedish bishop born in the seventeenth century [62]. Finally, we included three 1,000-year-old sequences, obtained from Peruvian human skeletons, closely related to extant *Mycobacterium pinnipedii* strains [57]. In the first two ancient datasets (Hungarian and Swedish), the myco pipeline was used as described above. However, the Peruvian dataset did not meet the predefined myco quality standards, i.e. having less than 20% of positions with a coverage below 10, and thus was discarded by the pipeline. As a result, a different variant-calling pipeline was used to analyse these sequences. In short, the pipeline calls the variants using the VarScan and Mutect2 callers [63, 64] and performs a variant consensus with Minos [25]. The final variants we use for placement have a depth of at least 20 reads and a minimum base quality of 15. Then, the output is converted to VCF using the faToVcf tool from the UShER toolbox and finally to MAPLE format using a custom script (https://github.com/lilymaryam/Mtb_global_phylogeny), so it can be placed with usher-sampled. After the placement of the six ancient samples, we used IQ-TREE to further reconstruct a local phylogeny of the monophyletic clade where these samples were placed to evaluate whether the placement performed by usher-sampled in a global context was able to retrieve the expected phylogenetic relationships seen at a local scale, also for ancient DNA samples.

## Results

### The myco bioinformatic pipeline for processing MTBC whole-genome sequences

We developed a comprehensive platform for genotyping and phylogenetic analysis of sequenced MTB genomes. This resource includes a bioinformatic pipeline written in WDL available from Dockstore (https://dockstore.org/workflows/github.com/aofarrel/myco/myco_sra; https://dockstore.org/workflows/github.com/aofarrel/myco/myco_raw) and GitHub (https://github.com/aofarrel/myco). A comprehensive visual of this workflow is found in Fig. S7, and information about using it can be found in the documentation on its GitHub repo and the Code Availability section. The pipeline is designed to be flexible for those who wish to reproduce our results while also being useful to public health officials who wish to use WGS data for outbreak analysis. As is typical for a WDL workflow, each task of the myco workflow has a runtime attributes section where it requests a certain amount of disc space, cores and memory from the backend, and different combinations of backends and executors may respect or ignore these runtime attributes depending on the specifics (https://cromwell.readthedocs.io/en/stable/RuntimeAttributes/) (https://miniwdl.readthedocs.io/en/latest/runner_reference.html#task-runtime-attributes). To represent a typical use case, we ran a test subset of 100 NCBI SRA samples on a Linux cluster to calculate the average execution time of the myco pipeline, obtaining a running time of 38.64 min (CI 95% 36.19–41.11). The running time needed to analyse each sample is detailed in Table S12. Users running the pipeline on similar setups or cloud compute platforms such as Terra will see similar runtimes. By default, the current version of myco requests 8 cores and 32 GB RAM for decontamination and filtering, 8 cores and 16 GB RAM for TBProfiler and 16 cores and 32 GB RAM for the variant caller. A low-resource mode is also available with a boolean toggle. The VCF obtained can be then uploaded to UShER-TB or processed locally, and the samples are placed in the global phylogeny in seconds.

### A comprehensive whole-genome MTBC phylogeny

MTBC is a worldwide respiratory pathogen of high public health importance. There are hundreds of thousands of available whole-genome sequences. As a consequence of technological limitations, these have not been aggregated into a single comprehensive phylogenetic tree. To meet that need, we considered all paired-end samples categorized as MTBC available on the SRA at the time (June 2024), representing 242,430 samples. Taking into account both manual identification of problematic samples and automated quality-checking via the myco pipeline, as well as removal of some miscategorized samples and samples incompatible with our pipeline, we generated TBProfiler and variant information for 135,180 samples (Tables S3 and S13.xlsx) and inferred a phylogenetic tree. We pruned 137 *M*. *canettii* samples (Table S4.xlsx), removed 5,631 through the FRS screening (see Methods) (Table S6.xlsx) and removed 100 more from manual inspection (Table S8.xlsx), leaving a total of 129,312 samples on the final phylogenetic tree. The succession of QC steps and the size of the dataset at each step are fully visualized in Fig. S8. The global phylogeny is encoded in a protocol buffer binary file [14] that is currently 17 Mb in size. To test placement efficiency, we randomly selected 100 samples from the dataset, duplicated them and added them to the tree as new samples. With 40 designated threads, this addition took 3 min and 11 s and used a peak of 7.1 GB of RAM.

### Rich metadata for the global phylogeny

Accurate and rich sample metadata is the most impactful resource for improving the utility and interpretability of genomic datasets and databases [65]. We developed Ranchero, a pip-installable Python library for obtaining standardized metadata for diverse samples. Using this tool, we processed all 242,430 BioSamples tagged as belonging to the *Mycobacterium* genus as of September 2024. In addition to standardizing NCBI SRA metadata fields for the entire genus, Ranchero parsed additional sample metadata from a variety of publications [26, 40, 43, 58, 66–75] (Table S2), which includes information not present in some samples' NCBI metadata fields, such as sampling locations and dates. This includes experimentally derived pDST data (Table S14) for 31,552 samples. The resulting genus-wide metadata file is provided as Table S3. This rich metadata ensures that the UShER-TB tree is interpretable for a wide variety of applications in public health, biomedical and evolutionary research aimed at understanding and controlling TB transmission.

A total of 71,741 (55.48%) of the 129,312 MTBC samples placed on the final phylogenetic tree are annotated with a host organism, the most common (*n*=66,827, 93.15%) being human, while 3,687 (5.14%) were labelled as cattle/bovine. The next most common hosts were badger (*n*=382, 0.53%), deer (*n*=306, 0.43%, including elk but excluding antelope) and pig/boar (*n*=176, 0.25%), respectively. Additionally, 49.69% (*n*=64,250) of samples are accompanied by metadata that identifies the year of isolation (Table S24).

In addition to collecting SRA metadata, Ranchero includes TBProfiler and general QC information for samples processed by our myco pipeline. From this, we determine that our samples typically had a median coverage above 50 (median=53, mean=52.37). Out of the 129,312 samples on our phylogenetic tree, TBProfiler assigned lineages to 99.88% (*n*=129,162) samples. TBProfiler sometimes applies more than one lineage per sample; a total of 128,960 samples were given a single unambiguous main lineage by TBProfiler. Out of these unambiguous lineages, the most commonly assigned was lineage 4 (*n*=57,109, 44.28% of data with one lineage), followed by lineage 2 (*n*=38,864, 30.13%), lineage 1 (*n*=11,442) and lineage 3 (*n*=11,300). The most common of the three animal-adapted lineages that TBProfiler can assign was La1 (*M. bovis*, *n*=8,276), followed by La3 (*M. orygis*, *n*=268) and La2 (*M. caprae*, *n*=184). TBProfiler additionally assigned 489 and 967 as the *M. africanum* lineages 5 and 6, respectively. The rarer lineage 7 (*n*=39), lineage 9 (*n*=27) and lineage 8 (*n*=3) were all present as well. This information is also present in the genus-level metadata file (Table S3), along with lineages provided in NCBI metadata tags and/or existing publications. TBProfiler additionally provided drug-resistance metadata for all samples (*n*=129,314) on the tree, which is labelled separately from the subset of data for which we have pDST. The most common drug resistance delineation from TBProfiler was Sensitive at 51.47% (*n*=66,561), followed by MDR-TB (indicating multiple drug resistance) at 18.25% (*n*=23,595). Notably, 11.95% (*n*=15,447) of samples were marked as ‘Other’, more than Pre-XDR-TB (*n*=11,688), HR-TB (9,255), RR-TB (2,412) and XDR-TB (356) delineations, showing the limitations of commonly used shorthands when describing drug resistance.

### Availability of the comprehensive UShER-TB resources

The final tree (Fig. 2a) and associated sample metadata (standardized by Ranchero and partially summarized in Fig. 2b) are available in several formats for visualization and sample placement. The 129,312 samples represent global coverage with the highest representation from Europe (37,662 samples) and Asia (31,569), led by the UK (14,724), China (9,780) and India (7,083), though geographic representation varies considerably due to differences in sequencing resources and surveillance capacity across regions. We developed a Taxonium build to host the tree for visualization and inspection of sample metadata (available from https://taxonium.org [38]). We also host the tree for download in various formats as a part of the UCSC Genome Browser (https://hgdownload.gi.ucsc.edu/hubs/GCF/000/195/955/GCF_000195955.2/UShER_Mtb_SRA). Additionally, as part of the UShER.bio project, users can place newly genotyped samples on the phylogenetic tree on demand at no cost (https://usher.bio).

**Fig. 2.**
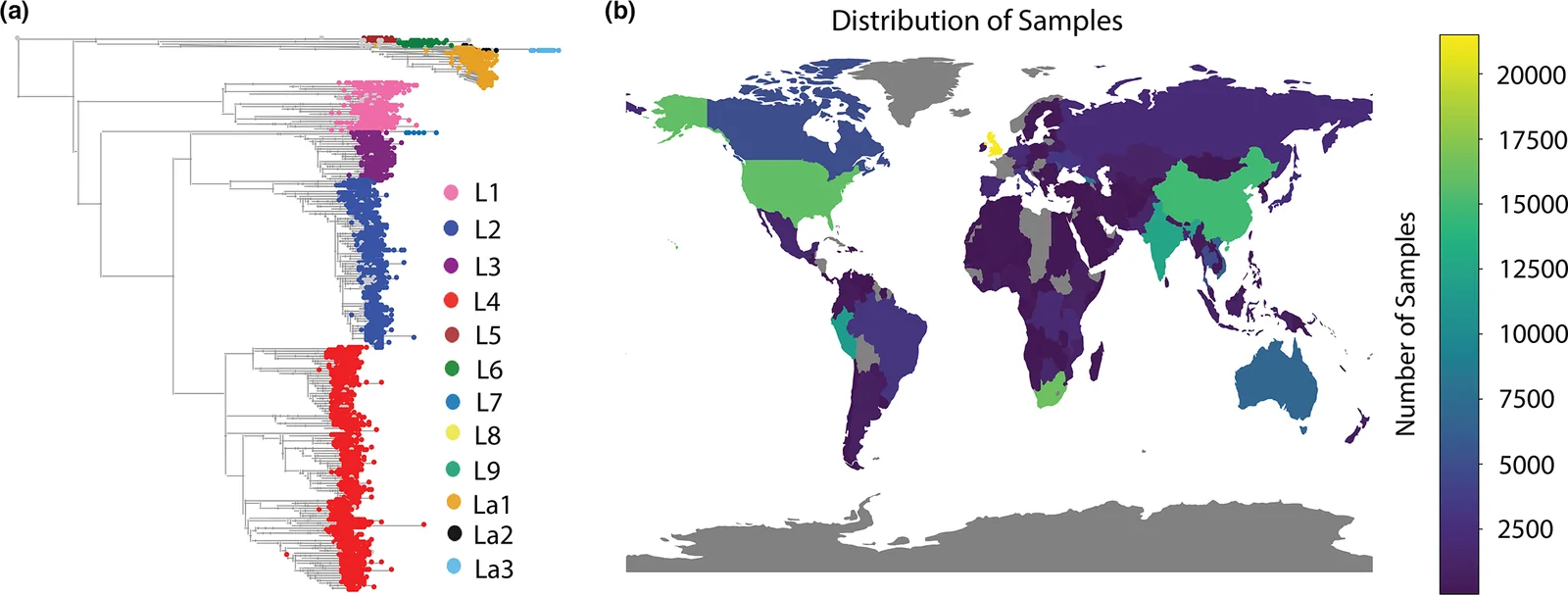
Summary of samples included in the global UShER-TB phylogenomics resource. (a) Global phylogeny of 129,312 samples processed with the myco pipeline and placed with usher-sampled. The phylogeny, samples and metadata can be explored interactively on Taxonium. (b) Geographical distribution of samples. Note that sample depth does not necessarily correspond to density of infections, as sequencing resources contribute heavily to data collection.

### Evaluation of phylogenetic inference accuracy and utility

To assess whether UShER-TB’s placement accuracy is sufficient for this MTBC resource to be used for detailed public health investigations into local outbreaks, we benchmarked the approach. One common analysis goal is to identify samples that are closely related to newly sequenced genomes [76–78]. We simulated data with properties similar to the global dataset (see Methods) and computed the ‘neighborhood recovery rate’ (hereafter NRR; see Methods), a metric that asks what proportion of samples within a given divergence time are discovered using a particular SNP-distance threshold. As expected, a larger SNP threshold can recover larger subsets of closely related samples (Fig. S3), and this NRR can be quite sensitive. For example, with a true divergence time of 1 year or less, a threshold of one SNP is able to recover all closest relatives in 83.2% of cases (Fig. S3). Therefore, a relatively small SNP threshold can accurately identify the closest relatives and will minimize the number of additional samples considered. Nonetheless, when new samples are obtained from geographic regions with little genomic data and correspondingly few close relatives are discovered, a larger threshold may be necessary.

In addition to this fine-scale NRR metric, we find that usher-sampled is able to accurately estimate the molecular evolutionary history of the species – i.e. the phylogenetic tree. The normalized Robinson–Foulds distance between the true and inferred phylogeny is 0.02 (where 0 would indicate identical topologies, and 1 completely discordant phylogenies). We also calculated RF between a phylogeny inferred by IQ-TREE2 and UShER-TB for the three real-world datasets enriched in transmission cases used in the later sections. For the Valencia Region, the normalized RF distance is 0.04; for Thailand, it is 0.01; and for Moldova, it is 0.11. All three values are quite modest, indicating good topological agreement between inference methods. This indicates that phylogenetic relationships are usually reconstructed by usher-sampled for real MTB genomic datasets. More generally, we conclude that these resources will be a valuable addition to the public health and evolutionary genomics toolkits for investigating MTBC evolution and transmission.

### Post phylogenetic inference processing of locally missing data

Missing data can significantly hinder accurate phylogenetic reconstruction for genomic datasets (see Methods; Fig. 3a). In public health settings, where SNP thresholds are often used to identify closely related samples, it’s crucial to exclude positions where a sample lacks a confident genotype. A common approach is to mask alignment positions with missing data in some samples. However, with the large number of samples in our analysis, this would result in masking nearly the entire genome.

**Fig. 3.**
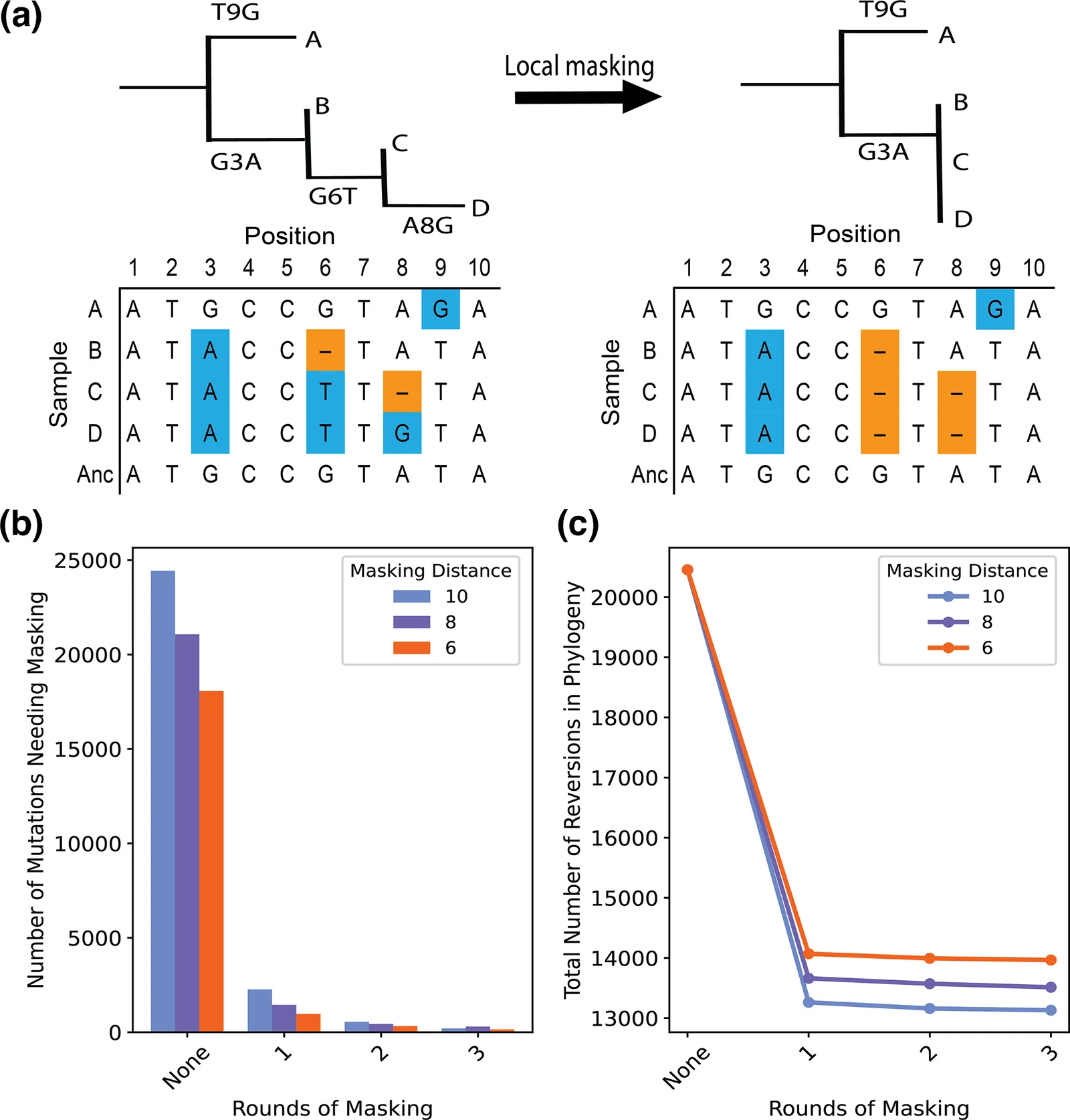
Motivation and effects of local masking. (a) Example demonstrating a placement artefact that occurs when samples have missing sequence information, where their close neighbours may have variants. On the left, Sample B has missing data (highlighted in the sequence alignment in orange) at position 6, while neighbours C and D share a >T mutation (mutations highlighted in blue) there. The placement algorithm treats this as a difference, inflating mutation distances despite alignment evidence. To avoid such false distances between closely related samples, we mask mutations like those at positions 6 and 8 (right) (see Methods). (b) To test the effect of multiple rounds of post-inference local masking, we tracked the number of mutations needing to be masked across four rounds of the procedure. (c) We tracked the number of revertant mutations present in the global tree after each round of masking to assess improvements in phylogenetic quality.

To address this, we developed a method to only mask locally missing data. Rather than masking sites across all samples, we only mask positions in a given sample if a closely related sample on the tree also lacks a confident genotype (see Methods; Fig. 3a). Because masking can alter the tree and introduce new missing data patterns, we applied multiple rounds of this local masking after phylogenetic inference to see if further improvements could be made. In each round, we measured the number of mutations that needed to be masked and the number of revertant mutations (mutations that mutate back to the ancestral allele) [79]. Although revertant mutations can occur naturally, the occurrence of reversions within a genomic dataset is more likely due to assembly or sequencing error (a sign of inaccurate phylogenetic reconstruction) [79].

After just one round of local masking, the number of mutations to be masked dropped by 91–94%, depending on the SNP distance threshold used (between 6 and 10). Revertant mutations also dropped by 41–45% under the same conditions. Using an SNP distance of 10, we masked 24,436 SNPs across 15,946 nodes and found that a single round was sufficient to identify the majority of problematic sites (Fig. 3b, c).

Notably, 11.2% of the masked sites contained revertant mutations – compared to just 0.63% of all mutations across the tree – suggesting that our approach effectively removes inaccurately inferred sites (Fig. 3c).

### Transmission cluster assignment in local and global datasets

We benchmarked usher-sampled against a standard pipeline for identifying transmission clusters based on pairwise distances using a 10-SNP threshold. Considering both approaches, we obtained a unique list of 769 samples in transmission at a maximum of 10 SNPs, of which 705 (91.7%) are shared in both methods. Considering the number of transmission clusters, we obtained a unique list of 230 clusters, 176 out of 230 (76.5%) that match between both methods. Out of the 54 non-matching clusters, 35 out of 230 (15.2%) only show 1 or 2 samples of difference between both methods, and most discrepancies can be corrected by simple visual inspection of the phylogenetic topology and branch lengths. The remaining 19 out of 230 (8.3%) clusters were only identified by the standard pipeline. Finally, we also calculated the precision, recall and adjusted Rand index to provide further insight on cluster assignment robustness of the new method against the gold standard, obtaining a precision of 0.98, a recall of 0.92 and an adjusted Rand index of 0.95. Cluster files from the standard pipeline and those obtained after using usher-sampled are available at https://github.com/lilymaryam/Mtb_global_phylogeny.

Having established that usher-sampled is accurate enough to identify most transmission clusters, we further explored usher-sampled’s utility for reconstructing genomic transmission clusters using real data from different settings and in different analysis scenarios, tailored by public health needs. Clustering is challenging, as it depends on low SNP thresholds (typically from 0 to 10) [80], and thus, any change in the analysis pipeline may affect the results. In our case, we estimated transmission clusters based on phylogenetic distances (see Methods) after *de novo* building reference phylogenies for each dataset.

As diverse transmission rates in the different settings may influence transmission cluster reconstruction, we estimated the proportion of samples in transmission for each dataset using a 10-SNP threshold. In the Valencian Region, Chiang Rai province and Moldova, transmission measured as genomic clustering rates accounted for 46.5% (*n*=716/1,540, 193 clusters), 37.1% (*n*=433/1,167, 119 clusters) and 62.3% (*n*=1,210/1,925, 245 clusters), respectively.

Considering this variety of settings, we evaluated the accuracy of placement for usher-sampled. At a local scale, i.e. mimicking ongoing regional surveillance, usher-sampled placed new samples with high accuracy and showed strong agreement with the original clusters. When using a global phylogenetic framework, the tool maintained cluster integrity while combining local and global context, further validating its robustness. Finally, in a global phylogeny with no previous genomic context, which simulates a real-world scenario of recently established genomic surveillance programmes, usher-sampled showed a high concordance with the original clusters.

Despite the concordance in clustering reconstruction among the different case scenarios, we observe a certain variation in the agreement between the local and global approaches. In the Valencian Region, the Cohen’s Kappa index is higher at a local level (0.993) than when we put the dataset in a global context (0.962). According to the cluster size and origin of the samples in these clusters, the proportion of small clusters (between 2 and 5 samples) is higher at a local level (78.4%) when compared to the global phylogeny (70.4% when placing 100% of the dataset). Additionally, most clusters now include lineage 4 samples from other countries, which is predominant in the region. In Thailand, we observe an increase in Cohen’s Kappa between the local and the global context, from 0.973 to 0.989. In this case, the proportion of small datasets remains virtually the same, decreasing from 89.3% to 87.7%, and a few clusters include samples from other countries. Finally, in Moldova, we observe a greater increase in the Cohen’s Kappa index, from 0.857 at a local level to 0.944 with a global context. The clusters in this case show a strong mixture of Moldovan samples with others from neighbouring countries like Ukraine, Russia or Georgia, and the proportion of small clusters decreases from 78% to 71.3%. Small cluster proportion in each case is described in Table S15, whereas the files containing the geographical origins of the samples can be found in the Git repository.

Overall, across all regions and situations, the agreement index doesn’t change remarkably, and the tool reliably positioned new samples near their original locations and preserved transmission group assignments (Fig. 4a). Cluster assignment for all samples in every case can be checked in Table S16.

**Fig. 4.**
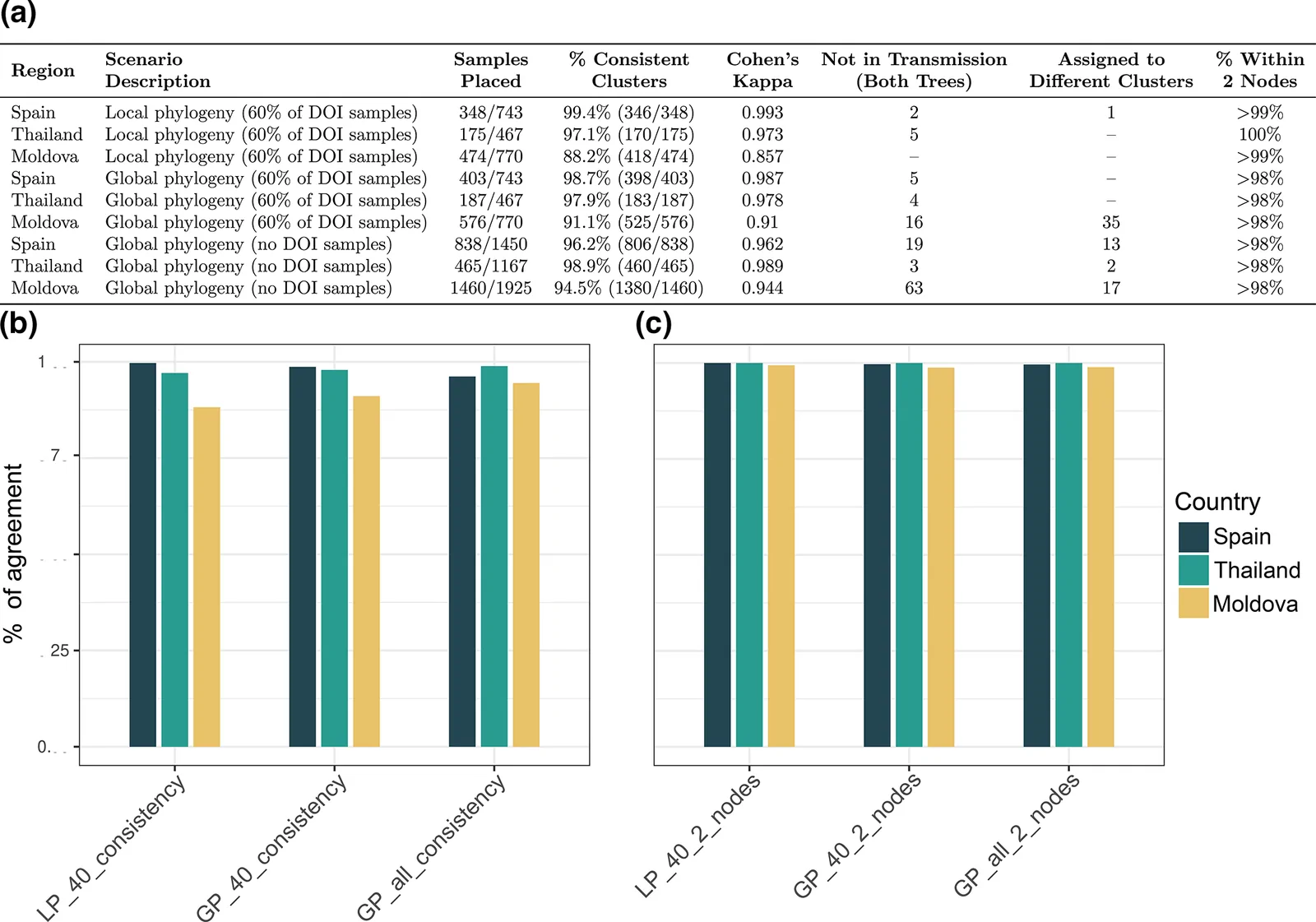
Placement consistency in different settings. (a) Summary of transmission cluster assignment results in local and global settings. (b) Proportion of samples that are correctly assigned to their original transmission group. (c) Proportion of samples that fall within two or less nodes from their original location in the tree. In both cases, different scenarios are considered. From left to right: local dataset placing 40% of samples (LP_40), global dataset placing 40% of the samples (GP_40) and global dataset placing all the sequences (GP_all).

### Cluster assignment is robust to local masking and recovers well at moderate FRS thresholds

To evaluate the sensitivity of 10-SNP cluster assignments to FRS pruning and local masking, we measured the NRR for 500 randomly selected samples across 15 FRS thresholds and 6 masking distances relative to the baseline tree (see Methods). We find that local masking distance has no effect on neighbourhood recovery across any FRS condition, consistent with our expectation that local masking refines placement of only a small number of samples without restructuring the tree (Fig. S1). FRS threshold is the dominant parameter: recovery improves steeply between FRS 0.05 and 0.15, then begins to plateau at ~95% mean recovery around FRS 0.16–0.17, with only marginal gains at higher thresholds.

### Demonstration of applicability for global epidemiological spread

A key strength of comprehensive phylogenomic resources such as this is the capacity to illuminate dynamics of global pathogen spread. In particular, we can track the frequency of pathogen lineage introductions into new geographic regions, which requires genomic sequences with accompanying geographic metadata. We investigated the utility of our resources for this purpose by calling new lineage introductions using a pandemic-era tool [46, 59]. We identified 51,077 total introductions, which we subsequently categorized as ‘singletons’ (where only one sample supports an introduction event into a given region) and ‘multiples’ (where two or more samples in a given region are closely related and likely to descend from a single introduction, potentially indicating ongoing transmission after introductions) (Fig. S4). This analysis revealed distinct introduction patterns among lineages, which are presumably related to the geographic endemic regions of each lineage and the geographic origin of the migrant flows related to each country but are also likely influenced by biases in sequencing efforts.

We investigated the robustness of epidemiological inferences using this full dataset by explicitly considering the biases of uneven sampling across geographic regions. A sensitivity analysis across 48 downsampling scenarios (see Methods) demonstrated that introduction patterns were stable in well-sampled countries but variable in regions with limited genomic representation (Figs S5 and S6). Therefore, we urge caution in interpreting analyses using UShER-TB and in particular for regions with lower sampling rates. These results highlight the utility of UShER-TB for identifying global transmission dynamics in adequately sampled regions and underscore the importance of continued genomic sampling efforts to improve inference in underrepresented areas.

### Identification of new candidate phylogenetically distinct clades

The scale of the global phylogeny is such that it may allow for the discovery and characterization of new phylogenetically distinct clades. To illustrate this potential, we present several noteworthy examples of samples that warrant further investigation. By creating a comprehensive global phylogeny, we can carefully inspect samples across the tree that have not been successfully assigned lineage metadata (Fig. 5). Among these unlabeled samples, we have found several that carry unique genetic variation not present in more widespread lineages. Sample SAMEA7423516 is sister to lineage 5 with a branch length (432) (diagnostic SNPs available in Text S3) comparable to the branch lengths of the samples within lineage 5 [two-sided MWU, *P*~0.15 (see Methods)] and passed all of our QC filters, including those intended to eliminate mixed infection samples. Sample SAMEA3448377 appears among the most genetically divergent samples in lineage 3, potentially representing a previously unknown sublineage. This Malawian sample has 353 unique mutations (Text S4) that distinguish it from its MRCA with other samples within lineage 3. We found no significant difference (*P*~0.09, Mann–Whitney U Test) between the branch length of SAMEA3448377 and sister samples in lineage 3, showing that SAMEA3448377 was not basally placed in lineage 3 and therefore is unlikely to be a mixed infection. Furthermore, the sample passed our QC filter for mixed bases.

**Fig. 5.**
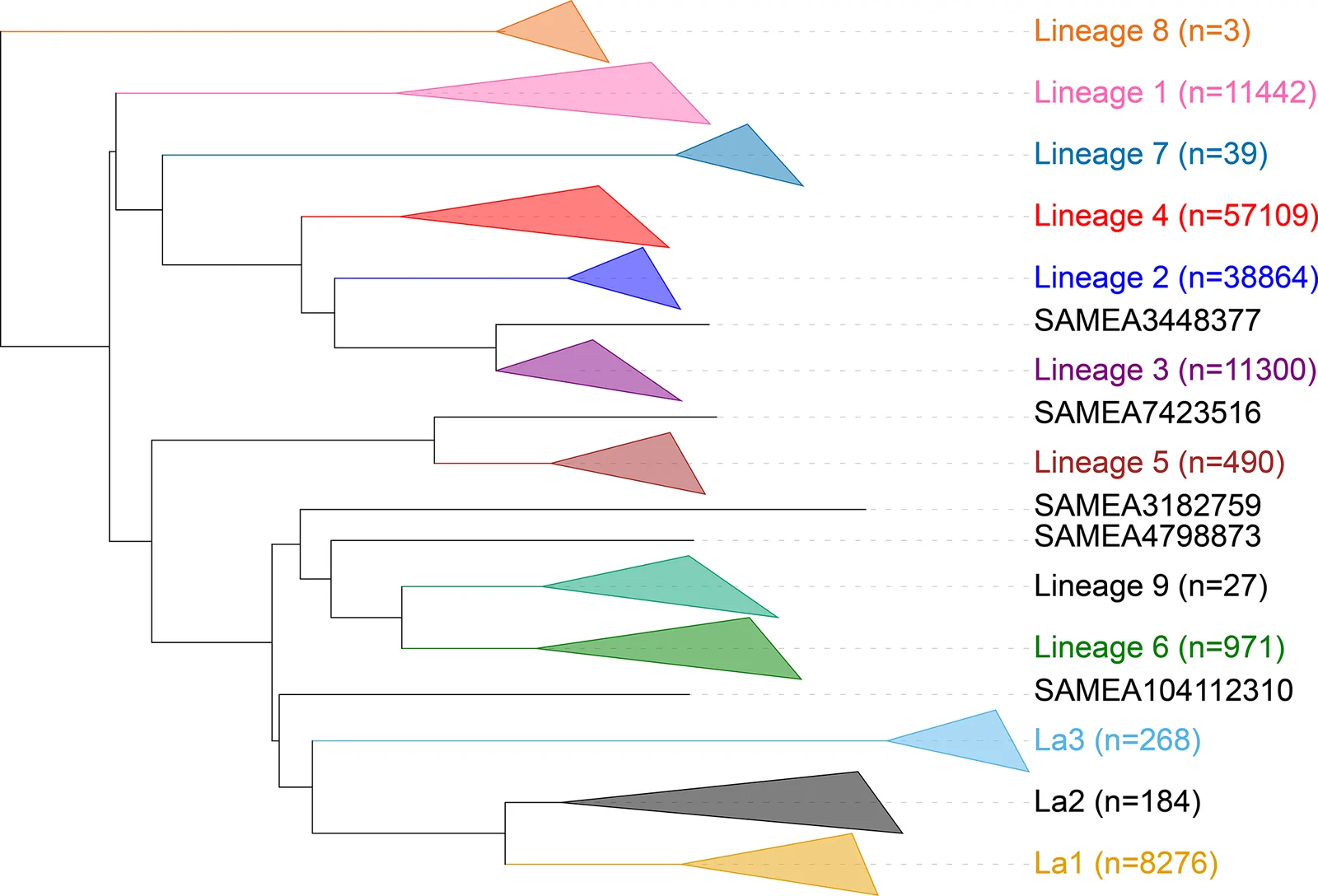
Location of candidate phylogenetically distinct clades. Summary of the UShER-TB global tree (with major lineages collapsed), illustrating where samples with potential novel clade designation are phylogenetically placed.

Several samples, including SAMEA3182759 and SAMEA4798873, are paraphyletic outgroups to lineages 6 and 9. These samples likely belong to a previously characterized animal-adapted MTBC clade A1 [40] (diagnostic SNPs available in Text S5 and S6). SAMEA104112310 is part of a small clade that is sister to the animal-hosted lineages La1–La3. This group of samples, including SAMEA104112310, appears to be part of an animal-hosted clade A2 that includes infections characterized as *M. microti* (land-mammal-hosted) and *M. pinnipedii* (pinniped-hosted) [40] (diagnostic SNPs available in Text S7).

To rule out placement uncertainty as a possible explanation for the distinct placement of these samples, we identified their maximum parsimony value and the number of equally parsimonious placements for each sample (Text S8). Quantification of the optimal placement positions measures the uncertainty conditional on the remainder of the phylogeny. In each case, the placements are highly certain (SAMEA7423516: Table S17; SAMEA3448377: Table S18; SAMEA3182759: Table S19; SAMEA4798873: Table S20; SAMEA104112310: Table S21), providing further support for the phylogenetic distinctness of each. These and other samples outside of major lineages demonstrate the utility of our global phylogenetic resource for comprehensively documenting the vast genetic variation across MTBC.

### Phylogenetic placement of ancient DNA pathogen samples

Current techniques have paved the way to sequence DNA from strains infecting individuals that lived hundreds of years ago. This allows us to better calibrate the evolution of the bacteria and the disease, improving our knowledge of how it emerged, how it evolved and its epidemiology in the past. UShER-TB is capable of assigning ancient DNA samples to their correct background. For example, MTB samples from mummies sampled in Hungary (SAMEA2810262 and SAMEA2810266) are placed in nodes enriched in L4.1.2.1 and L4.8/L4.10, respectively (Fig. 6a, b), matching their original lineage typing [61]. An MTB genome obtained from a 17th Century Swedish Bishop [62] (SAMN14570812) is also assigned to the correct L4.8/L4.10 clade (Fig. 6c). As for genomes from Peruvian pre-contact human skeletal remains, they are thought to be close [57] to an animal clade related to *M. pinnipeddii*, associated with seals and sea lions. UShER-TB, however, places the samples in a basal position to the common clade between *M. pinnipedii* and *M. microti* sequences, instead of closer to *M. pinnipeddii* (Fig. 6d). After reconstructing a maximum-likelihood tree with IQ-TREE containing the strains of the monophyletic node in which the new samples are placed, we retrieved the expected topology for this region of the phylogeny as published in Bos *et al*. [57] (Fig. S9). The example illustrates that analysis of most ancient DNA samples is technically challenging and will need a tailored pipeline, which is beyond the scope of this study. Nonetheless, our results show that UShER-TB can perform initial screening of the samples and genotyping and identify their phylogeographic background for detailed downstream analyses.

**Fig. 6.**
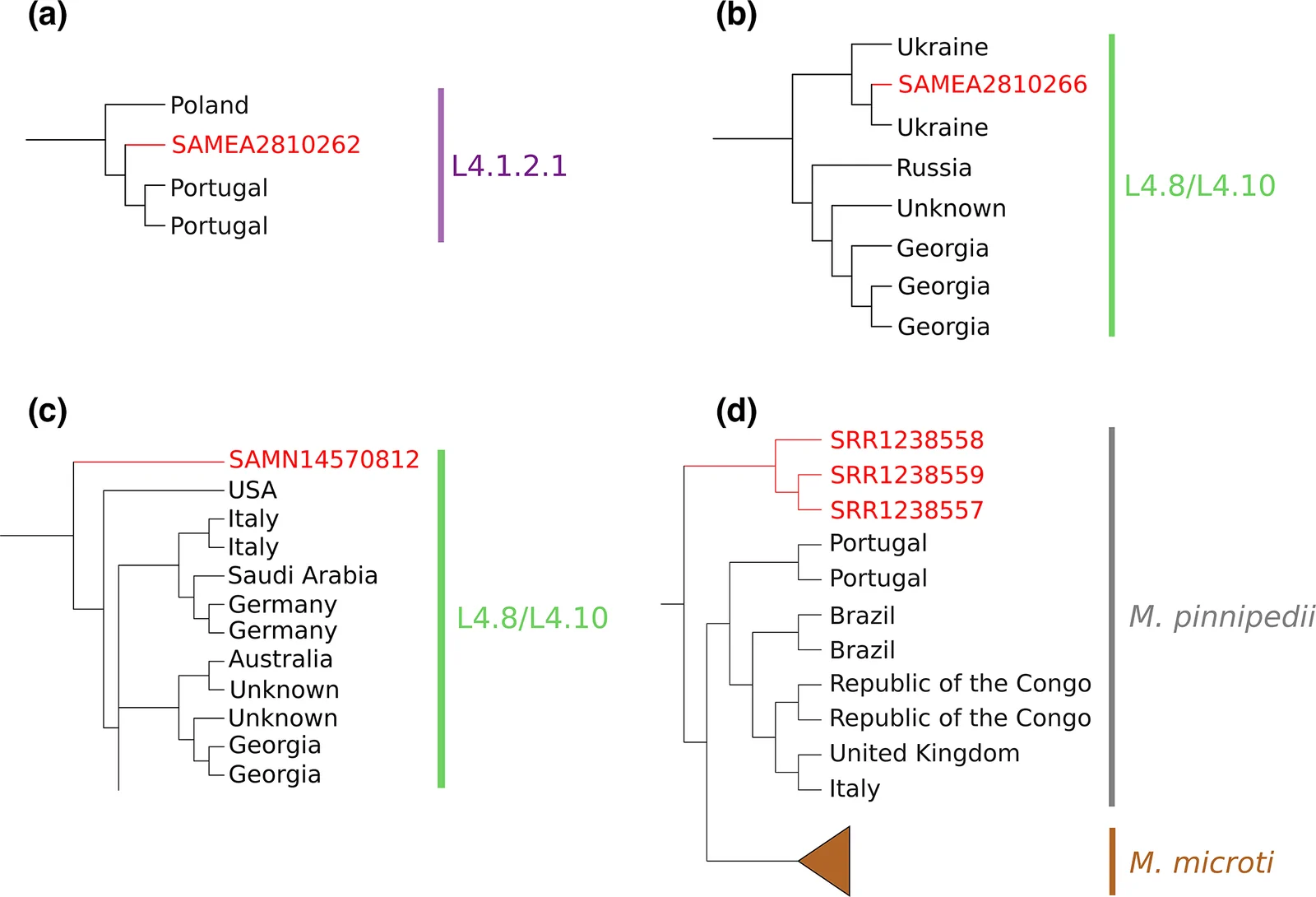
Phylogenetic neighbourhoods of ancient DNA-placed samples. All the nodes show the placed samples (in red), the neighbouring taxa codified by the country in which the samples were collected and the lineage of the samples within the node. (a) and (b) correspond to the Hungarian mummies’ sequences, (c) corresponds to the Bishop’s sequence and (d) shows the *M. pinnipedii* sequences.

## Discussion

This study establishes a globally comprehensive genomic resource for MTB, addressing long-standing challenges in TB phylogenetics and transmission inference. By leveraging large-scale publicly available sequencing data and implementing efficient tree-building methodologies, this work substantially increases the scale at which MTB genomes can be analysed. The ability to rapidly place new genomes into a global phylogeny provides a more nuanced understanding of TB transmission dynamics, helping to distinguish between recent and historical transmission events with greater confidence. These improvements have practical implications for public health, particularly in settings where genomic surveillance has been constrained by computational limitations or data fragmentation.

While UShER-TB provides a powerful framework for real-time phylogenetic analysis of MTB, several important limitations must be considered when interpreting results. These span technical, epidemiological and genomic dimensions of the approach.

Bioinformatics pipeline variation can introduce technical artefacts that affect placement accuracy. We recommend that new samples be processed using uniform bioinformatics pipelines, as differences in variant calling, quality filtering or read mapping approaches may introduce artefacts that affect phylogenetic placement accuracy and comparability.

Uneven global sampling creates substantial challenges for phylogeographic interpretation. Sampling remains highly non-uniform globally, with particularly sparse representation from high-burden regions in Africa and other low-resource settings. Such sampling variation has several important consequences for inference. Observed lineage distributions may reflect sampling intensity rather than epidemiological reality, with lineages appearing over-represented in well-sampled regions (e.g. Europe and North America) while appearing rare or absent in under-sampled settings where they may actually circulate widely. Sparse sampling also artificially inflates introduction counts by rendering local transmission chains invisible; what appear as multiple independent introductions may represent a single introduction event with unsampled intermediates, and under-sampled regions may disproportionately appear as sources rather than recipients of transmission. Additionally, genomic clusters fragment when key samples are missing, causing genuinely connected cases to appear as singletons or small isolated clusters and systematically underestimating transmission chain connectivity in under-sampled regions.

Users should be cautious when interpreting phylogenetic placements and transmission patterns for samples from underrepresented regions. Phylogenetic placement on long branches may reflect sampling gaps rather than evolutionary novelty, and nearest-neighbour matches may be geographically and temporally distant from true relatives; genetic distances should be treated as upper bounds. Apparent transmission directions from well-sampled to poorly-sampled regions may be reversed when local diversity is unsampled, and singleton or small cluster assignments likely indicate missing transmission chain members rather than true epidemiological isolation. We recommend that users from high-burden, undersampled settings interpret placements as hypothesis-generating rather than definitive and prioritize building regional reference datasets to improve contextual resolution.

Samples from undersampled regions require careful interpretation. Long phylogenetic branches typically reflect gaps in sampling rather than genuine evolutionary divergence. The closest genetic match in the tree may be geographically or temporally distant from the sample’s true relatives, so these distances represent upper bounds on actual genetic relationships. Sampling gaps can also distort transmission inference: a region may appear to export cases when it is actually receiving them, simply because its local diversity is missing from the tree. Samples that appear isolated or form small clusters often represent unsampled transmission chains rather than true epidemiological singletons. For users in high-burden settings with limited representation in global datasets, phylogenetic placements should be treated as preliminary findings rather than definitive evidence. Building regional reference datasets remains the most effective way to improve placement accuracy and transmission inference.

Our results show that UShER-TB is able to correctly reconstruct transmission clusters by placing new sequences in an existing phylogeny in different contexts: using local datasets, using global datasets with previous genomic context and with no context at all. However, the inclusion of global context seems to be beneficial in settings with high lineage diversity like Thailand or strong cross-border transmission, as we observe in the Moldovan dataset, since it provides the missing transmission links, usually with neighbouring countries. However, in settings with localized outbreaks like Valencia, adding global context may add noise to the cluster resolution.

Our current approach focuses exclusively on SNPs and does not capture structural genomic variation, including large insertions, deletions or rearrangements, as well as regions inaccessible to short-read mapping that may significantly impact MTB phenotypes such as antibiotic resistance [81–83]. Future integration with pangenome approaches (such as Walia *et al*. [84]) and an increasing use of long-read technologies may help address this limitation by incorporating structural variation into comprehensive phylogenomic analyses [85, 86]. Likewise, our ancient DNA analysis, while a proof of concept, suggests that additional adjustments in the analysis pipeline of the samples are needed to incorporate those challenging samples into UShER-TB phylogeny. These considerations underscore the importance of continued efforts to expand global genomic surveillance and to refine analytical methodologies for MTB.

Beyond its immediate epidemiological applications, this resource also enhances broader research efforts into TB evolution, drug resistance emergence and phylogeographic patterns. By integrating a vast number of genomes into a single analytical framework, this approach minimizes biases introduced by selective subsampling and allows for more representative insights into MTB population structure. Moreover, its computational efficiency ensures that researchers in a wide range of settings, including those with limited resources, can access and utilize these tools effectively [87]. Notably, the dataset and framework provided by UShER-TB enable small-scale analyses against global context, which was previously impossible for lower resource labs with fewer, local samples. While additional barriers remain in many countries – such as access to stable internet connections and others involving major investments in education and technology [18, 19] – tools like UShER-TB will help to ameliorate the limited access to bioinformatics skills and resources in the Global South. As TB control strategies increasingly incorporate genomic data, the methods and dataset presented here provide a foundation for more comprehensive and timely investigations into the spread and adaptation of this persistent pathogen.

## Supplementary material

10.1099/mgen.0.001859 Supplementary Material 1.

## Abbreviations

FRS: fraction of read support
MAPLE: MAximum Parsimonious Likelihood Estimation
MAT: Mutation Annotated Tree
MRCA: most recent common ancestor
MTB: *Mycobacterium tuberculosis*
MTBC: *Mycobacterium tuberculosis* complex
NRR: neighbourhood recovery rate
pDST: phenotypic drug susceptibility testing
QC: quality control
SRA: Sequence Read Archive
TB: tuberculosis
UShER: ultrafast sample placement on existing tRees
VCF: Variant Call Format
WDL: workflow development language

## References

[1] Karim LM, Martínez-Martínez FJ, O’Farrell A, Hinrichs AS, Sanderson T (2026). *Figshare*.

[2] Hershkovitz I, Donoghue HD, Minnikin DE, May H, Lee OY-C (2015). Tuberculosis origin: the neolithic scenario. Tuberculosis.

[3] Paulson T (2013). Epidemiology: A mortal foe. Nature.

[4] World Health Organization (2023). Global tuberculosis report 2023. https://www.who.int/publications/i/item/9789240083851.

[5] World Health Organization (2026). The top 10 causes of death. https://www.who.int/news-room/fact-sheets/detail/the-top-10-causes-of-death.

[6] Davies PDO (2003). The role of DOTS in tuberculosis treatment and control. Am J Respir Med.

[7] Ogden J, Walt G, Lush L (2003). The politics of “branding” in policy transfer: the case of DOTS for tuberculosis control. Soc Sci Med.

[8] Dye C, Glaziou P, Floyd K, Raviglione M (2013). Prospects for tuberculosis elimination. Annu Rev Public Health.

[9] Farhat M, Cox H, Ghanem M, Denkinger CM, Rodrigues C (2024). Drug-resistant tuberculosis: a persistent global health concern. Nat Rev Microbiol.

[10] Goig GA, Loiseau C, Maghradze N, Mchedlishvili K, Avaliani T (2025). Transmission as a Key Driver of Resistance to the New Tuberculosis Drugs. N Engl J Med.

[11] Comas I, López MG, Chiner-Oms Á, Farhat MR, Ngabonziza JCS (2023).

[12] Goig GA, Windels EM, Loiseau C, Stritt C, Biru L (2025). Ecology, global diversity and evolutionary mechanisms in the Mycobacterium tuberculosis complex. *Nat Rev Microbiol*.

[13] De Maio N, Kalaghatgi P, Turakhia Y, Corbett-Detig R, Minh BQ (2023). Maximum likelihood pandemic-scale phylogenetics. Nat Genet.

[14] Turakhia Y, Thornlow B, Hinrichs AS, De Maio N, Gozashti L (2021). Ultrafast Sample placement on Existing tRees (UShER) enables real-time phylogenetics for the SARS-CoV-2 pandemic. Nat Genet.

[15] Hinrichs A, Ye C, Turakhia Y, Corbett-Detig R (2024). The ongoing evolution of UShER during the SARS-CoV-2 pandemic. Nat Genet.

[16] Martínez-Martínez FJ, Massinga AJ, De Jesus Á, Ernesto RM, Cano-Jiménez P (2023). Tracking SARS-CoV-2 introductions in Mozambique using pandemic-scale phylogenies: a retrospective observational study. Lancet Glob Health.

[17] Lam-Hine T, McCurdy SA, Santora L, Duncan L, Corbett-Detig R (2021). Outbreak Associated with SARS-CoV-2 B.1.617.2 (Delta) Variant in an Elementary School - Marin County, California, May-June 2021. *MMWR Morb Mortal Wkly Rep*.

[18] Olono A, Mitesser V, Happi A, Happi C (2024). Building genomic capacity for precision health in Africa. Nat Med.

[19] Onywera H, Ondoa P, Nfii F, Ogwell A, Kebede Y (2024). Boosting pathogen genomics and bioinformatics workforce in Africa. Lancet Infect Dis.

[20] NCBI https://www.ncbi.nlm.nih.gov/sra.

[21] Voss K, Gentry J, Van der Auwera G (2017). Full-stack genomics pipelining with GATK4 + WDL + Cromwell. Epub ahead of print 8.

[22] Terra https://app.terra.bio/.

[23] Github.

[24] https://github.com/iqbal-lab-org/clockwork.

[25] Hunt M, Letcher B, Malone KM, Nguyen G, Hall MB (2022). Minos: variant adjudication and joint genotyping of cohorts of bacterial genomes. Genome Biol.

[26] The CRyPTIC Consortium (2022). A data compendium associating the genomes of 12,289 Mycobacterium tuberculosis isolates with quantitative resistance phenotypes to 13 antibiotics. PLoS Biol.

[27] Li H (2018). Minimap2: pairwise alignment for nucleotide sequences. Bioinformatics.

[28] Danecek P, Bonfield JK, Liddle J, Marshall J, Ohan V (2021). Twelve years of SAMtools and BCFtools. *GigaScience*.

[29] Iqbal Z, Caccamo M, Turner I, Flicek P, McVean G (2012). De novo assembly and genotyping of variants using colored de Bruijn graphs. Nat Genet.

[30] Bolger AM, Lohse M, Usadel B (2014). Trimmomatic: a flexible trimmer for Illumina sequence data. Bioinformatics.

[31] seqtk LH https://github.com/lh3/seqtk.

[32] Marin M, Vargas R, Harris M, Jeffrey B, Epperson LE (2022). Benchmarking the empirical accuracy of short-read sequencing across the M. tuberculosis genome. Bioinformatics.

[33] https://github.com/iqbal-lab-org/cryptic_tb_callable_mask.

[34] UCSC Genome Browser BedGraph track format. https://genome.ucsc.edu/goldenpath/help/bedgraph.html.

[35] Danecek P, Auton A, Abecasis G, Albers CA, Banks E (2011). The variant call format and VCFtools. Bioinformatics.

[36] Phelan JE, O’Sullivan DM, Machado D, Ramos J, Oppong YEA (2019). Integrating informatics tools and portable sequencing technology for rapid detection of resistance to anti-tuberculous drugs. Genome Med.

[37] Coll F, McNerney R, Preston MD, Guerra-Assunção JA, Warry A (2015). Rapid determination of anti-tuberculosis drug resistance from whole-genome sequences. Genome Med.

[38] Sanderson T (2022). Taxonium, a web-based tool for exploring large phylogenetic trees. eLife.

[39] Pfyffer GE, Auckenthaler R, Embden JD, Soolingen D Mycobacterium canettii, the smooth variant of m. Emerg Infect Dis.

[40] Brites D, Loiseau C, Menardo F, Borrell S, Boniotti MB (2018). A New Phylogenetic Framework for the Animal-Adapted *Mycobacterium tuberculosis* Complex. Front Microbiol.

[41] Supply P, Marceau M, Mangenot S, Roche D, Rouanet C (2013). Genomic analysis of smooth tubercle bacilli provides insights into ancestry and pathoadaptation of Mycobacterium tuberculosis. Nat Genet.

[42] Koeck J-L, Fabre M, Simon F, Daffé M, Garnotel E (2011). Clinical characteristics of the smooth tubercle bacilli “Mycobacterium canettii” infection suggest the existence of an environmental reservoir. Clin Microbiol Infect.

[43] Coscolla M, Gagneux S, Menardo F, Loiseau C, Ruiz-Rodriguez P (2021). Phylogenomics of Mycobacterium africanum reveals a new lineage and a complex evolutionary history. *Microbial Genomics*.

[44] Ngabonziza JCS, Loiseau C, Marceau M, Jouet A, Menardo F (2020). A sister lineage of the Mycobacterium tuberculosis complex discovered in the African Great Lakes region. Nat Commun.

[45] Ye C, Thornlow B, Hinrichs A, Kramer A, Mirchandani C (2022). matOptimize: a parallel tree optimization method enables online phylogenetics for SARS-CoV-2. Bioinformatics.

[46] McBroome J, Thornlow B, Hinrichs AS, Maio N, Goldman N (2021). A daily-updated database and tools for comprehensive SARS-cov-2 mutation-annotated trees. Epub ahead of print 4.

[47] Ly-Trong N, Naser-Khdour S, Lanfear R, Minh BQ (2022). AliSim: A Fast and Versatile Phylogenetic Sequence Simulator for the Genomic Era. *Mol Biol Evol*.

[48] Nei M, Kumar S (2000). Molecular Evolution and Phylogenetics.

[49] Robinson DF, Foulds LR (1981). Comparison of phylogenetic trees. *Mathematical Biosciences*.

[50] Kramer AM, Thornlow B, Ye C, De Maio N, McBroome J (2023). Online Phylogenetics with matOptimize Produces Equivalent Trees and is Dramatically More Efficient for Large SARS-CoV-2 Phylogenies than de novo and Maximum-Likelihood Implementations. Syst Biol.

[51] Turakhia Y, De Maio N, Thornlow B, Gozashti L, Lanfear R (2020). Stability of SARS-CoV-2 phylogenies. PLoS Genet.

[52] Menardo F, Gagneux S, Freund F (2021). Multiple Merger Genealogies in Outbreaks of Mycobacterium tuberculosis. Mol Biol Evol.

[53] Menardo F, Duchêne S, Brites D, Gagneux S (2019). The molecular clock of Mycobacterium tuberculosis. PLoS Pathog.

[54] Walker TM, Ip CLC, Harrell RH, Evans JT, Kapatai G (2013). Whole-genome sequencing to delineate Mycobacterium tuberculosis outbreaks: a retrospective observational study. Lancet Infect Dis.

[55] Guerra-Assunção JA, Crampin AC, Houben RMGJ, Mzembe T, Mallard K (2015). Large-scale whole genome sequencing of M. tuberculosis provides insights into transmission in a high prevalence area. Elife.

[56] Bryant JM, Schürch AC, van Deutekom H, Harris SR, de Beer JL (2013). Inferring patient to patient transmission of Mycobacterium tuberculosisfrom whole genome sequencing data. BMC Infect Dis.

[57] Bos KI, Harkins KM, Herbig A, Coscolla M, Weber N (2014). Pre-Columbian mycobacterial genomes reveal seals as a source of New World human tuberculosis. Nature.

[58] Cancino-Muñoz I, López MG, Torres-Puente M, Villamayor LM, Borrás R (2022). Population-based sequencing of *Mycobacterium tuberculosis* reveals how current population dynamics are shaped by past epidemics. Elife.

[59] McBroome J, Martin J, de Bernardi Schneider A, Turakhia Y, Corbett-Detig R (2022). Identifying SARS-CoV-2 regional introductions and transmission clusters in real time. Virus Evol.

[60] Walker TM, Lalor MK, Broda A, Ortega LS, Morgan M (2014). Assessment of Mycobacterium tuberculosis transmission in Oxfordshire, UK, 2007-12, with whole pathogen genome sequences: an observational study. Lancet Respir Med.

[61] Kay GL, Sergeant MJ, Zhou Z, Chan JZ-M, Millard A (2015). Eighteenth-century genomes show that mixed infections were common at time of peak tuberculosis in Europe. Nat Commun.

[62] Sabin S, Herbig A, Vågene ÅJ, Ahlström T, Bozovic G (2020). A seventeenth-century Mycobacterium tuberculosis genome supports a Neolithic emergence of the Mycobacterium tuberculosis complex. Genome Biol.

[63] Koboldt DC, Zhang Q, Larson DE, Shen D, McLellan MD (2012). VarScan 2: somatic mutation and copy number alteration discovery in cancer by exome sequencing. Genome Res.

[64] Benjamin D, Sato T, Cibulskis K, Getz G, Stewart C (2019).

[65] Huang Y-N, Munteanu V, Love MI, Ronkowski CF, Deshpande D (2025). Perceptual and technical barriers in sharing and formatting metadata accompanying omics studies. *Cell Genom*.

[66] Walker TM, Miotto P, Köser CU, Fowler PW, Knaggs J (2022). The 2021 WHO catalogue of *Mycobacterium tuberculosis* complex mutations associated with drug resistance: A genotypic analysis. *Lancet Microbe*.

[67] Stucki D, Brites D, Jeljeli L, Coscolla M, Liu Q (2016). Mycobacterium tuberculosis lineage 4 comprises globally distributed and geographically restricted sublineages. Nat Genet.

[68] Shuaib YA, Utpatel C, Kohl TA, Barilar I, Diricks M (2022). Origin and Global Expansion of *Mycobacterium tuberculosis* Complex Lineage 3. *Genes (Basel*).

[69] Nimmo C, Ortiz AT, Tan CCS, Pang J, Acman M (2024). Detection of a historic reservoir of bedaquiline/clofazimine resistance-associated variants in Mycobacterium tuberculosis. Genome Med.

[70] Napier G, Campino S, Merid Y, Abebe M, Woldeamanuel Y (2020). Robust barcoding and identification of Mycobacterium tuberculosis lineages for epidemiological and clinical studies. Genome Med.

[71] Menardo F, Rutaihwa LK, Zwyer M, Borrell S, Comas I (2021). Local adaptation in populations of *Mycobacterium tuberculosis* endemic to the Indian Ocean Rim. F1000Res.

[72] Menardo F, Loiseau C, Brites D, Coscolla M, Gygli SM (2018). Treemmer: a tool to reduce large phylogenetic datasets with minimal loss of diversity. BMC Bioinformatics.

[73] Finci I, Albertini A, Merker M, Andres S, Bablishvili N (2022). Investigating resistance in clinical Mycobacterium tuberculosis complex isolates with genomic and phenotypic antimicrobial susceptibility testing: a multicentre observational study. *Lancet Microbe*.

[74] Coll F, Phelan J, Hill-Cawthorne GA, Nair MB, Mallard K (2018). Genome-wide analysis of multi- and extensively drug-resistant Mycobacterium tuberculosis. Nat Genet.

[75] Andres S, Gröschel MI, Hillemann D, Merker M, Niemann S (2019). A Diagnostic Algorithm To Investigate Pyrazinamide and Ethambutol Resistance in Rifampin-Resistant Mycobacterium tuberculosis Isolates in a Low-Incidence Setting. *Antimicrob Agents Chemother*.

[76] Baker CR, Barilar I, de Araujo LS, Rimoin AW, Parker DM (2023). Use of High-Resolution Geospatial and Genomic Data to Characterize Recent Tuberculosis Transmission, Botswana. *Emerg Infect Dis*.

[77] Auld SC, Shah NS, Cohen T, Martinson NA, Gandhi NR (2018). Where is tuberculosis transmission happening? Insights from the literature, new tools to study transmission and implications for the elimination of tuberculosis. Respirology.

[78] Guthrie JL, Gardy JL (2017). A brief primer on genomic epidemiology: lessons learned from Mycobacterium tuberculosis. Ann N Y Acad Sci.

[79] Hunt M, Hinrichs AS, Anderson D, Karim L, Dearlove BL (2024). Addressing pandemic-wide systematic errors in the SARS-CoV-2 phylogeny. Bioinformatics.

[80] Meehan CJ, Moris P, Kohl TA, Pečerska J, Akter S (2018). The relationship between transmission time and clustering methods in Mycobacterium tuberculosis epidemiology. EBioMedicine.

[81] Jones RM, Adams KN, Eldesouky HE, Sherman DR (2022). The evolving biology of *Mycobacterium tuberculosis* drug resistance. Front Cell Infect Microbiol.

[82] Luna MJ, Oluoch PO, Miao J, Culviner P, Papavinasasundaram K (2025). Frequently arising ESX-1-associated phase variants influence *Mycobacterium tuberculosis* fitness in the presence of host and antibiotic pressures. mBio.

[83] Ates LS, Dippenaar A, Ummels R, Piersma SR, van der Woude AD (2018). Mutations in ppe38 block PE_PGRS secretion and increase virulence of Mycobacterium tuberculosis. Nat Microbiol.

[84] Walia S, Motwani H, Smith K, Corbett-Detig R, Turakhia Y (2024). Compressive Pangenomics Using Mutation-Annotated Networks. Bioinformatics.

[85] Stritt C, Reitsma M, Marin AMG, Goig G, Dötsch A (2025). Gene conversion and duplication contribute to genetic variation in an outbreak of Mycobacterium tuberculosis. *Microbial Genomics*.

[86] García-Marín AM, Torres-Puente M, Martinez-Priego L, De Marco G, Moreno-Molina M (2026). Complete genomes reveal a refined map of Mycobacterium tuberculosis genetic diversity across evolutionary scales. *Nat Commun*.

[87] Vasiliu A, Saktiawati AMI, Duarte R, Lange C, Cirillo DM (2022). Implementing molecular tuberculosis diagnostic methods in limited-resource and high-burden countries. *Breathe (Sheff*).

