# Supplementary material for: UShER-TB: scalable, comprehensive, accessible phylogenomic analysis of Mycobacterium tuberculosis
Source: Microb Genom. 2026 Oct 22;12(10):001859. doi: 10.1099/mgen.0.001859 (PMC13690021; doi:10.1099/mgen.0.001859)
Supplement: Supplementary file 1 [file mgen-12-01859-s001.pdf]

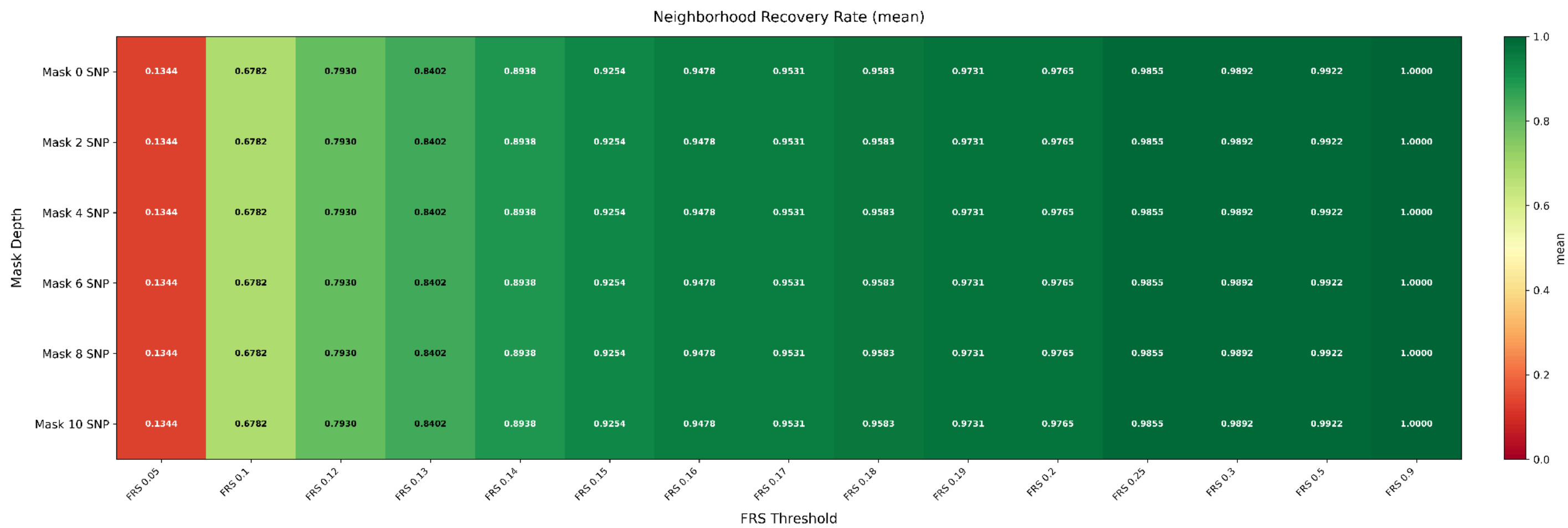

Supplemental Fig 1

Samples labeled by percentage of variants falling below FRS threshold (see Methods)

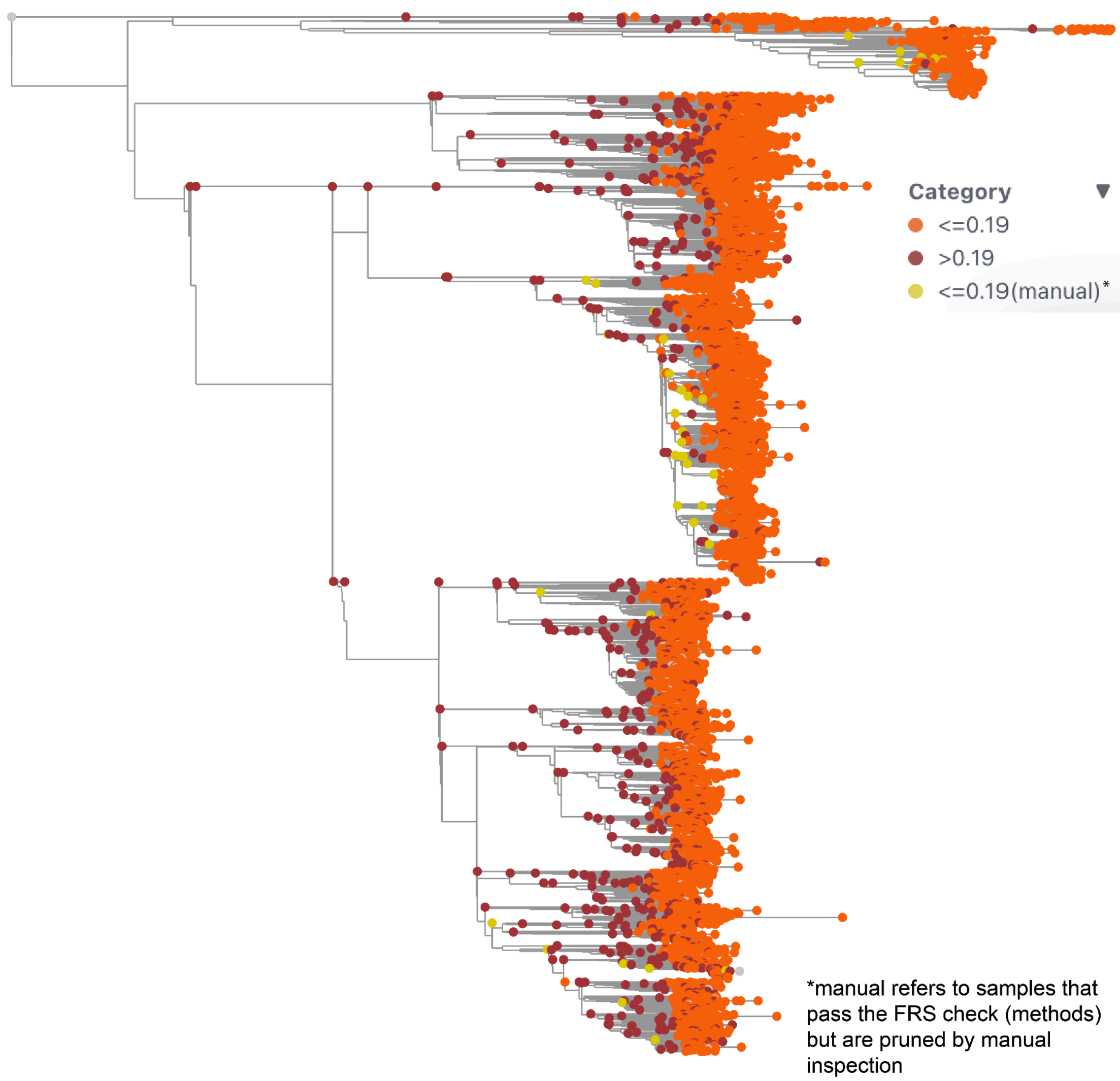

Supplemental Fig 2

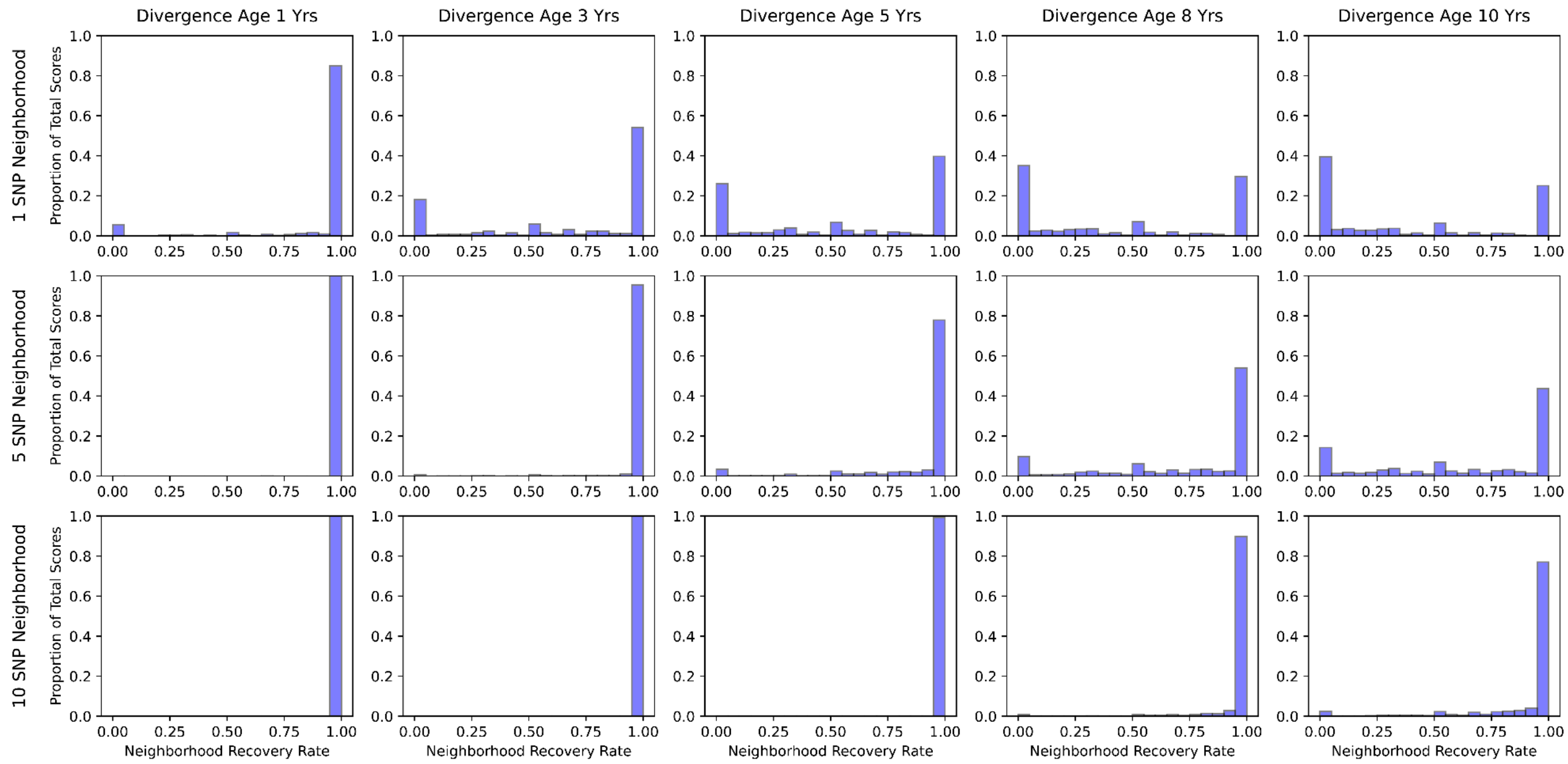

Supplemental Fig 3

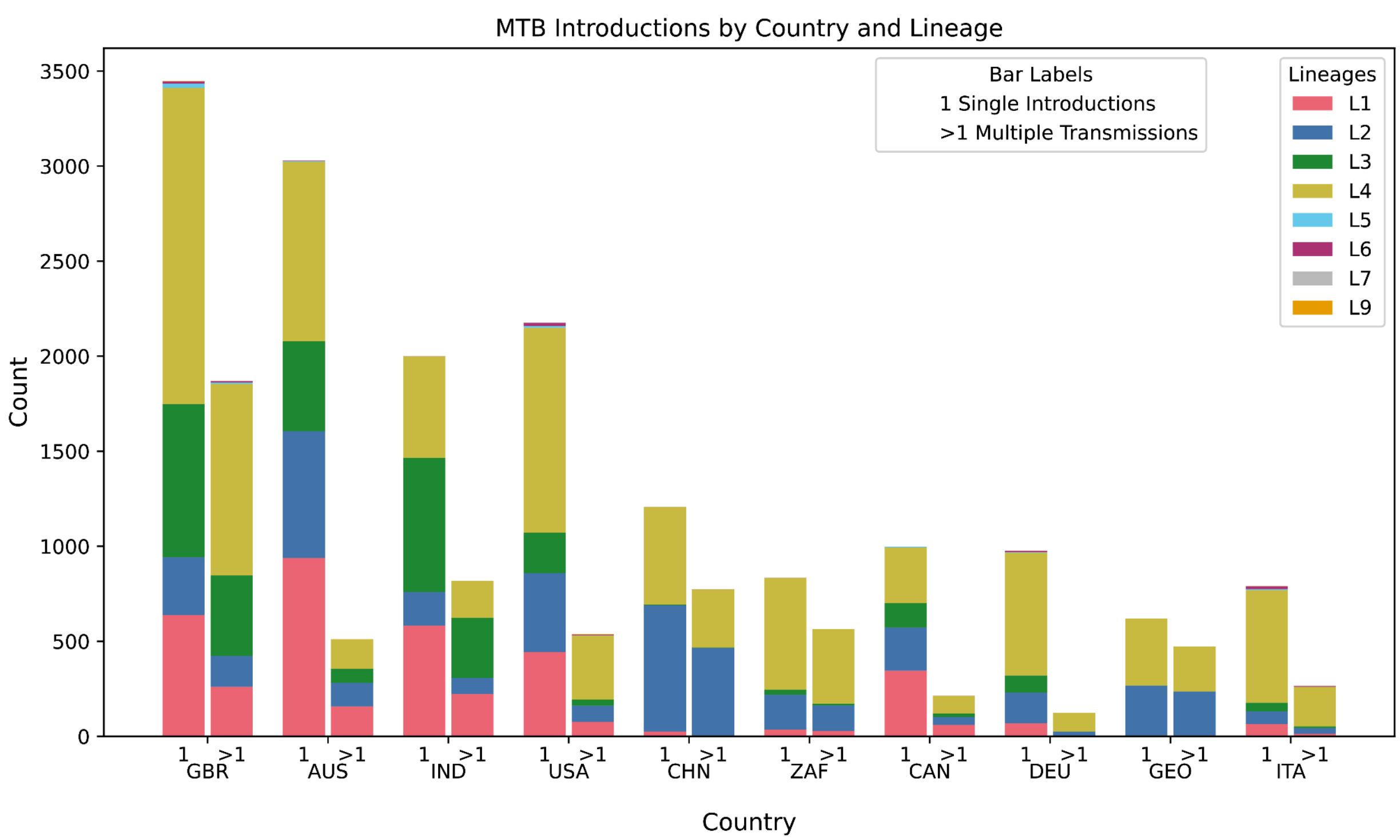

Supplemental Fig 4

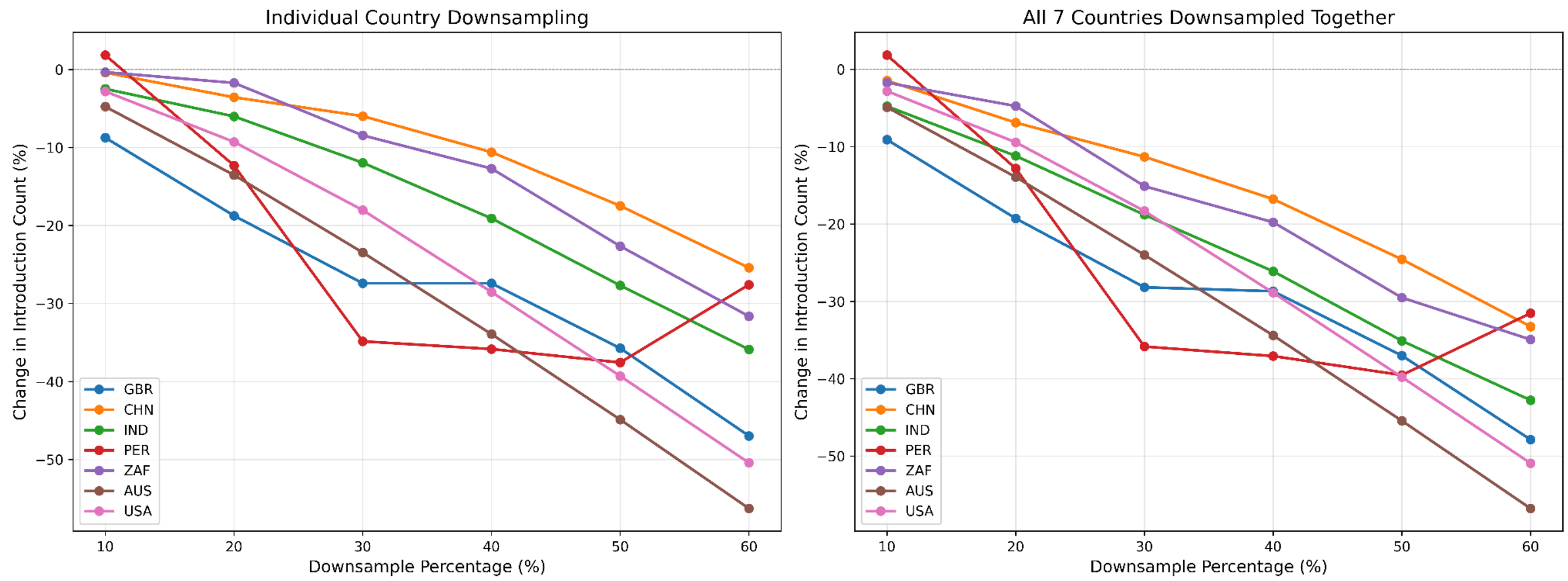

Supplemental Fig 5

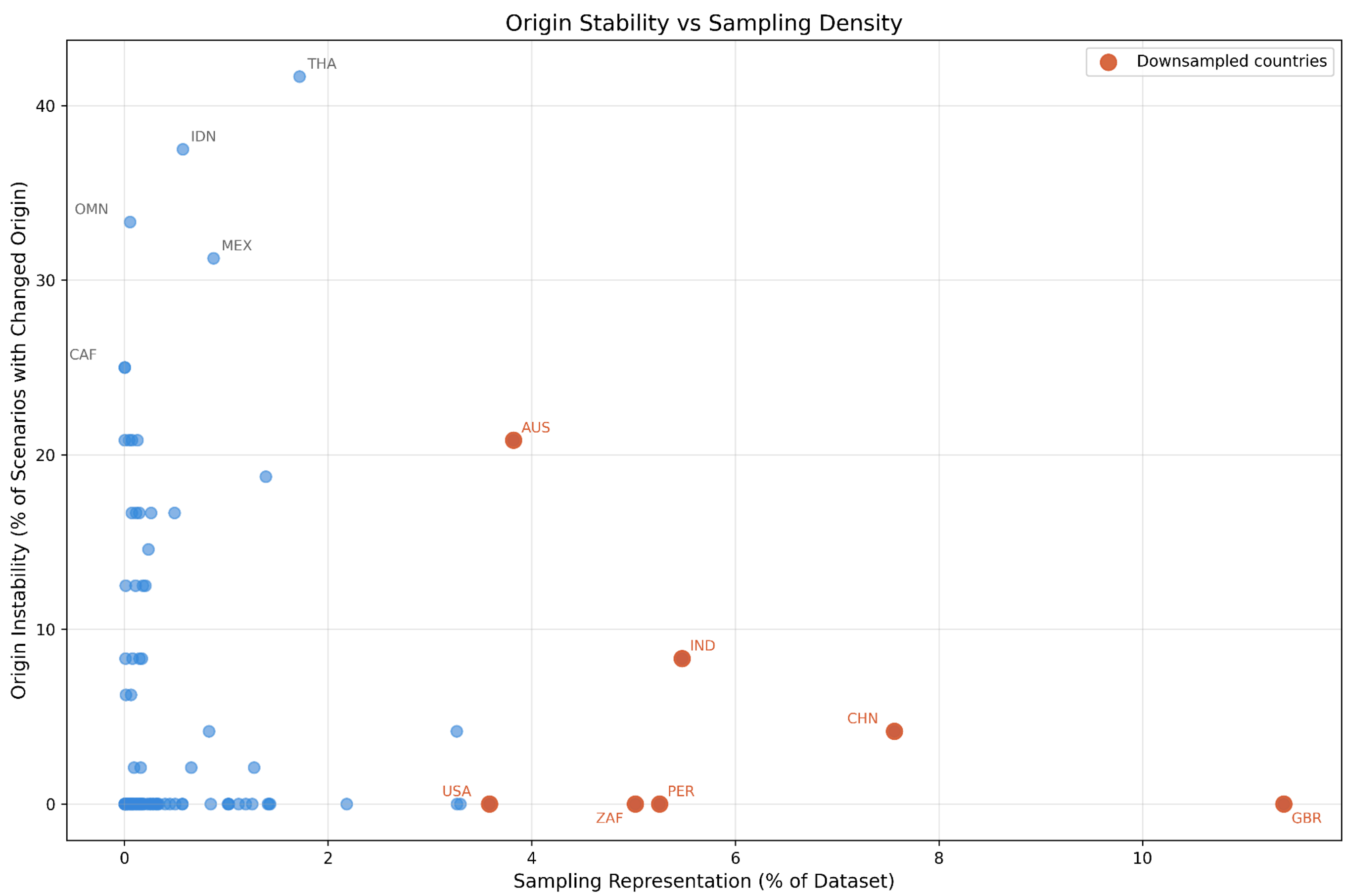

Supplemental Fig 6

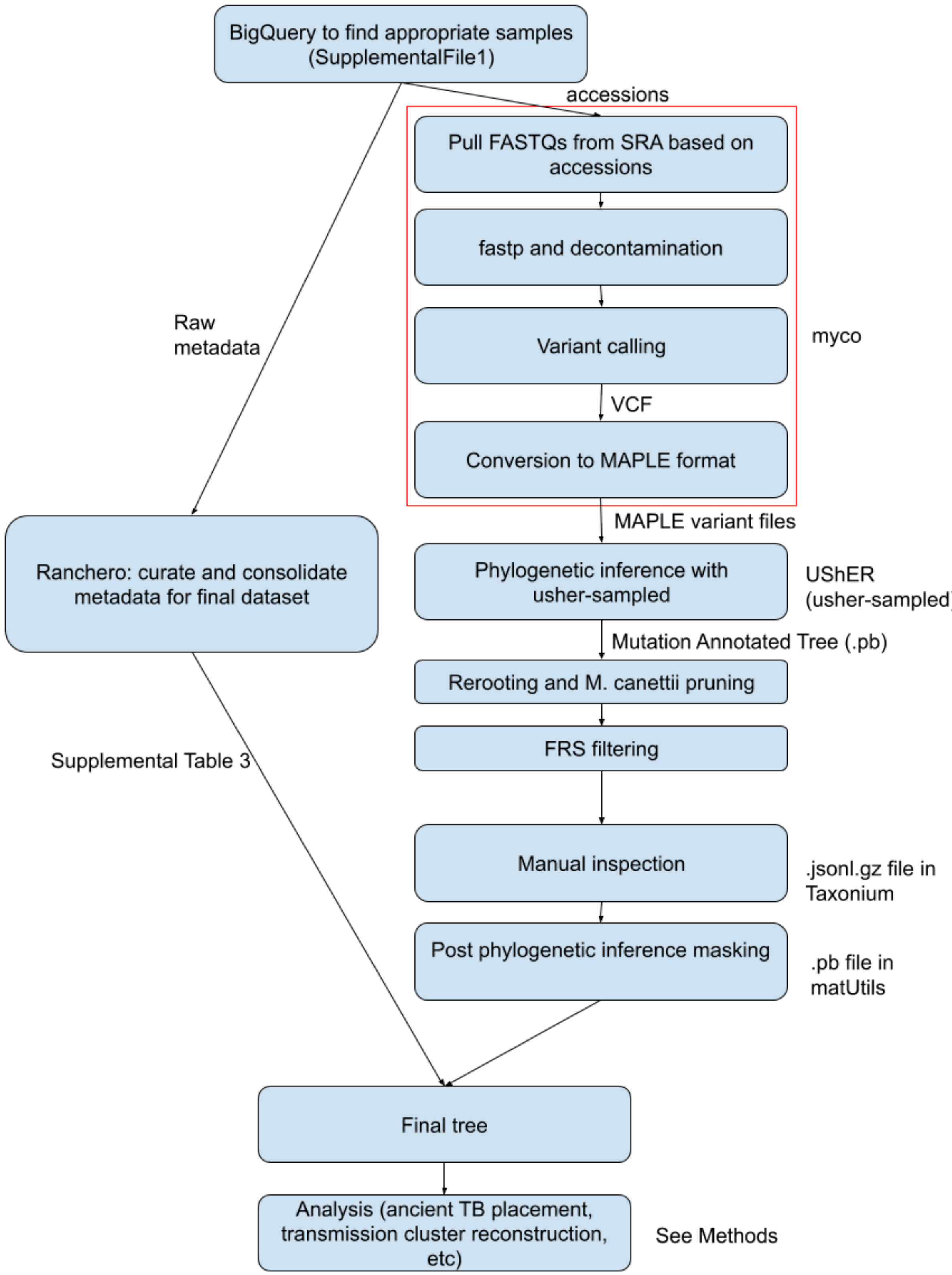

Supplemental Fig 7

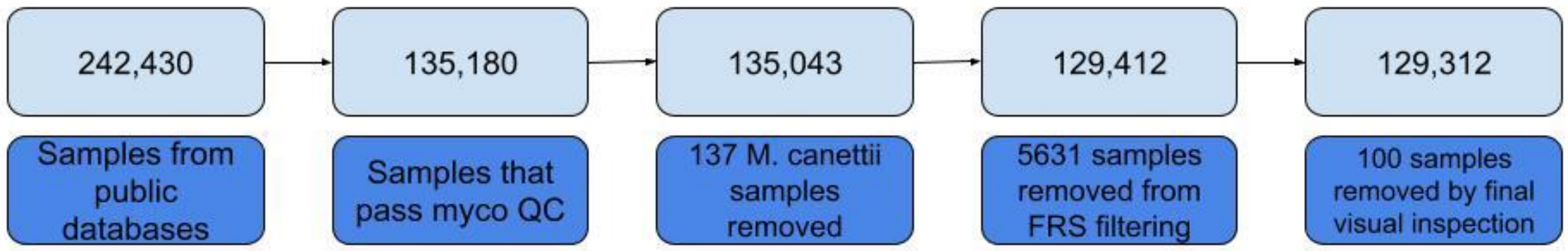

Supplemental Fig 8

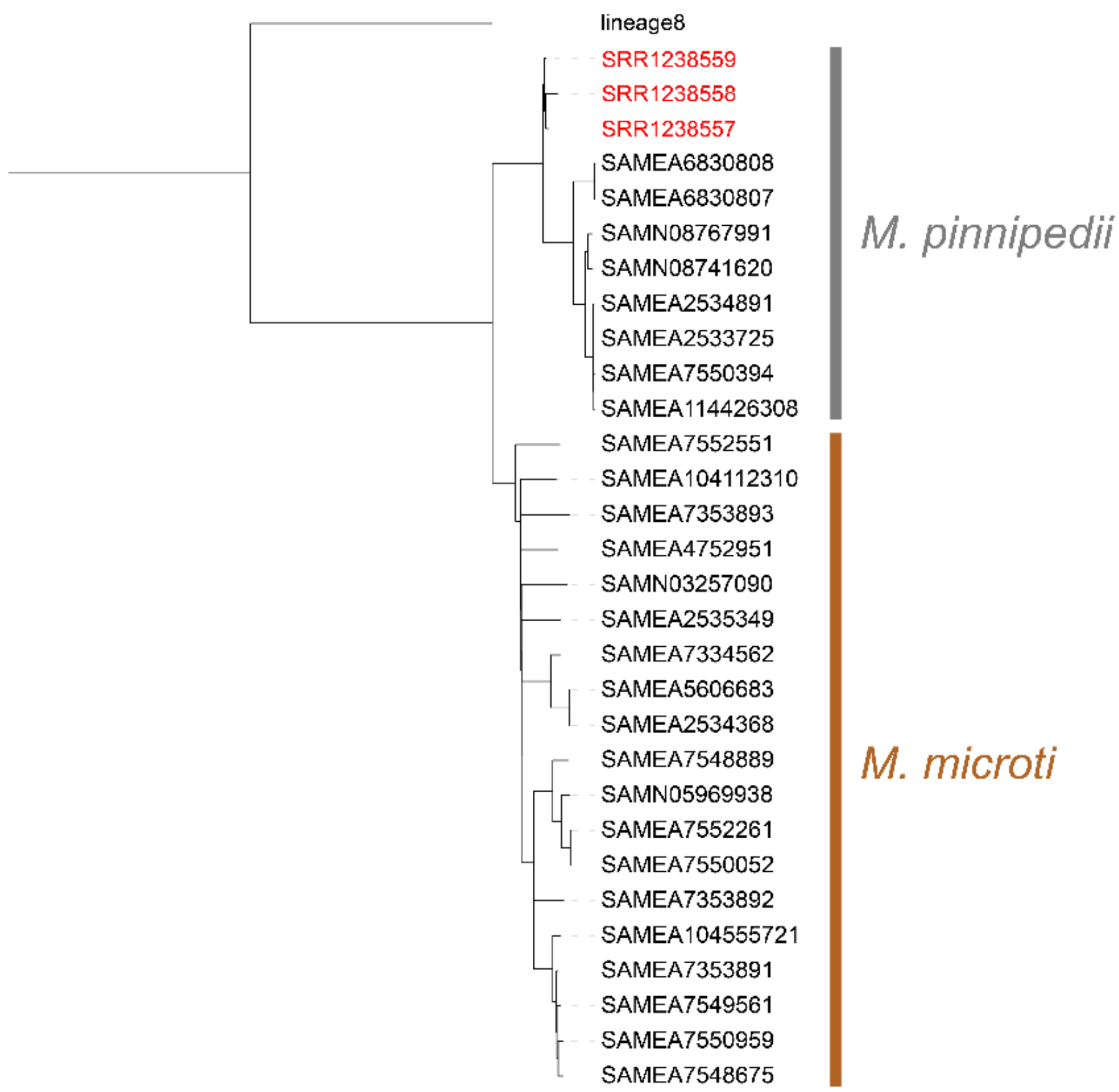

Supplemental Fig 9
